# Characterization of Dynamic Regulatory Gene and Protein Networks in Wheat Roots Upon Perceiving Water Deficit Through Comparative Transcriptomics Survey

**DOI:** 10.3389/fpls.2021.710867

**Published:** 2021-08-16

**Authors:** Yousef Rahimi, Pär K. Ingvarsson, Mohammad Reza Bihamta, Hadi Alipour, Alireza Taleei, Shaghayegh Khoshnoodi Jabar Abadi

**Affiliations:** ^1^Department of Plant Biology, Swedish University of Agricultural Sciences, Uppsala, Sweden; ^2^Department of Agronomy and Plant Breeding, Faculty of Agriculture, University of Tehran, Karaj, Iran; ^3^Department of Plant Production and Genetics, Faculty of Agriculture and Natural Resources, Urmia University, Urmia, Iran

**Keywords:** root development, wheat, water deficit, DEGs, functional analysis, PPI

## Abstract

A well-developed root system benefits host plants by optimizing water absorption and nutrient uptake and thereby increases plant productivity. In this study we have characterized the root transcriptome using RNA-seq and subsequential functional analysis in a set of drought tolerant and susceptible genotypes. The goal of the study was to elucidate and characterize water deficit-responsive genes in wheat landraces that had been through long-term field and biochemical screening for drought tolerance. The results confirm genotype differences in water-deficit tolerance in line with earlier results from field trials. The transcriptomics survey highlighted a total of 14,187 differentially expressed genes (DEGs) that responded to water deficit. The characterization of these genes shows that all chromosomes contribute to water-deficit tolerance, but to different degrees, and the B genome showed higher involvement than the A and D genomes. The DEGs were mainly mapped to flavonoid, phenylpropanoid, and diterpenoid biosynthesis pathways, as well as glutathione metabolism and hormone signaling. Furthermore, extracellular region, apoplast, cell periphery, and external encapsulating structure were the main water deficit-responsive cellular components in roots. A total of 1,377 DEGs were also predicted to function as transcription factors (TFs) from different families regulating downstream cascades. TFs from the AP2/ERF-ERF, MYB-related, B3, WRKY, Tify, and NAC families were the main genotype-specific regulatory factors. To further characterize the dynamic biosynthetic pathways, protein-protein interaction (PPI) networks were constructed using significant KEGG proteins and putative TFs. In PPIs, enzymes from the CYP450, TaABA8OH2, PAL, and GST families play important roles in water-deficit tolerance in connection with MYB13-1, MADS-box, and NAC transcription factors.

## Introduction

A well-developed root system is an essential component of plant productivity, as roots contribute to a host of plant growth functions (Khan et al., 2016). Roots function by anchoring plants to the soil but they also provide a platform for water and nutrient uptake and benefit plants by facilitating interactions with different symbiosis-forming micro-organisms in the rhizosphere (Comas et al., 2013; Fang et al., 2017). The spatial distribution of roots is referred to as the root system architecture (RSA) and this is known to dynamically change when encountering external elements (Lynch, 1995). An optimized RSA, both in terms of anatomy and growth attributes, can increase water absorption from different layers of soil and subsequently increase plant productivity (Wasson et al., 2012).

Despite difficulties in obtaining reliable root measurements, there have been attempts to study RSA which has resulted in a better understanding of differences in root system morphology between different species (Hochholdinger and Tuberosa, 2009; Bishopp and Lynch, 2015; Maccaferri et al., 2016). These studies suggest that plant roots generally comprise both embryo-formed roots, which include primary and seminal roots, and post embryonically-formed roots, including adventitious roots (Lynch and Brown, 2012; Khan et al., 2016). The growth features of these roots, such as elongation, longevity, and lateral dispersion are controlled by many genes and their interactions with the environment, as well as hormone homeostasis, play an important role in root development (Lynch and Brown, 2012; Smith and De Smet, 2012; Paez-Garcia et al., 2015). It has been suggested that auxin produced in both root and shoot affects the expression level of genes that are involved in cell division (Overvoorde et al., 2010). Cell-cycle genes, including Cyclin-dependent-kinases (CDKs) and D-type cyclin (CYCD), are activated by auxin and their suppression leads to reduced root branching (Nieuwland et al., 2009; Khan et al., 2016).

However, there is still a clear lack of genomic knowledge on roots compared to aboveground organs, although access to high throughput sequencing technologies, such as RNA-sequencing and previously microarrays has facilitated genomics-based comparative studies especially in model species and crop plants (Wang et al., 2009; Ma et al., 2017; Xiong et al., 2017). Wheat is one of these important crop species that has been feeding a considerable portion of humans for over 5,000 years (Peng et al., 2011). Meanwhile, around 50% of wheat arable lands are currently affected by drought and future climate change may increase it to more than 60% (Fahad et al., 2017; Hernandez-Ochoa et al., 2018; Pessarakli, 2019; Saddique et al., 2020).

Roots are integral to the detection networks for drought stress and this induce changes in hormone signaling, carbon allocation, protein, and carbohydrate metabolisms which, in turn, affects plant biomass production (Giehl et al., 2014; Paez-Garcia et al., 2015). A better understanding of the genomic regions controlling root development would therefore assist in identifying key biosynthetic pathways involved in root growth. In this paper we present the results of (I) biochemical and root morphology studies in wheat genotypes with contrasting drought response phenotypes, (II) perform comparative transcriptomics of root cells, (III) assess functional enrichment of water deficit-responsive biosynthetic pathways and regulatory elements, (IV) and create a protein-protein interaction network to elucidate underlying genomic loci and dynamic elements in root development under water deficit conditions.

## Materials and Methods

### Plant Materials and Water Treatments

A collection of 320 Iranian bread wheat accessions was evaluated during the 2016–17 and 2017–18 cropping seasons for a number of agronomic traits at the Agricultural Research Lands of the Department of Agronomy and Plant Breeding, University of Tehran (Rahimi et al., 2019). From this germplasm, 100 landraces were selected based on drought tolerance indices and their performance under drought conditions, ranging from susceptible to tolerant (Supplementary Table 1). By applying polyethylene glycol 6000 (PEG 6000) on seeds, we simulated water deficit conditions at the seedling growth stage. The growth parameters of seedlings assessed included fresh and dry matter, length of roots and shoot, percentage of germination and germination speed. The genotypes were also grown in the greenhouse and exposed to stress conditions [50% field capacity (FC)] 4 weeks after planting. Leaves samples were collected 1 week after exposure to stress conditions to study antioxidant capacity.

### Assessment of Hydrogen Peroxide (H_2_O_2_) and Malondialdehyde (MDA) Content

To measure H_2_O_2_ content, 0.40 g of fresh leaves were homogenized using 6 ml of 0.1% (w/v) trichloroacetic acid (TCA). Samples were centrifuged at 12,000 × g and 0.6 ml of the resulting supernatant was transferred to a 2 ml tube mixed with 0.5 ml of 10 mM phosphate buffer and 0.9 ml 1 M KI. The solutions were kept under dark condition for 1 h after which the absorbance of the solution at 390 nm was scored (Loreto and Velikova, 2001). To measure MDA, 0.2 g of fresh leaves were ground by adding 5 ml 0.1% TCA. After centrifuging the homogenate at 8,000 × *g* for 5 min, 1 ml of the resulting supernatant was pipetted into a new tube and mixed with 4.5 ml of 20% TCA. The solution was centrifuged at 4,000 × *g* for 10 min and the absorbance of the solution was read at 532 nm (Heath and Packer, 1968).

### Measurement of Proline Content and Superoxide Dismutase (SOD) Activity

To measure proline content we used a ninhydrin reaction buffer based on the method of Bates et al. (1973). The proteins of leaves were extracted using the Bradford (1976) method with a phosphate buffer (pH 7.0). The resulting supernatant was further used to determine soluble proteins and antioxidant enzyme activity. The activity of SOD was measured using the procedure from Dhindsa et al. (1982), where the enzyme activity is measured by its ability to inhibit photochemical reduction. An amount of SOD which inhibits the photochemical reduction of nitroblue tetrazolium by 50% is referred to one unit of the enzyme.

### Preparing RNA Samples

After field trials and *in-situ* studies, 19 landraces were selected, ranging from extremely susceptible to very drought tolerant. These landraces were then used for further investigations at the Swedish University of Agricultural Sciences, Uppsala, Sweden. Seeds of these genotypes were kindly provided by the International Maize and Wheat Improvement Center (CIMMYT). Isolated landraces were grown in the greenhouse by applying 16/8 h light and dark and were then exposed to either normal irrigation or water deficit conditions 4 weeks after planting. Roots of drought stressed-plants and their corresponding controls from the normal irrigation scheme were sampled at three time-points; 9, 18, and 25 days after applying stress (DA). Water stress treatment involved supplying no water to pots in the stress treatment while control pots were irrigated regularly every 4 days until sampling. At each time point, three biological replicates were collected for each treatment, resulting in a total of 342 samples that were evaluated for root attributes. Root length, root and shoot biomass and root/shoot ratio of all samples were recorded, and all samples were subsequently frozen in liquid nitrogen and stored at −70°C. After assessing grain yield under drought conditions, seedling traits, antioxidant activity, osmoprotectant capacity, and root features, three landraces, including two tolerant and one susceptible, were selected for RNA extraction. We tried to select genetically differentiated tolerant landraces to get insights into possible differences in biosynthesis pathways used under severe conditions. Overall, we used three landraces, PI627299 (tolerant), PI627038 (tolerant), and PI624837 (susceptible), two water treatments (normal irrigation and water deficit stress) and two time points (nine and 18 DA) with three biological replicates for a total of 36 unique sample combinations that were used for RNA extraction.

### RNA Isolation

Total RNA was extracted using a QIAGEN RNeasy®Plant Mini Kit. Briefly, 100 mg of plant material was disrupted in liquid nitrogen and transferred to an RNase-free 2 ml microcentrifuge tube. 450 μl of RLT Buffer was added and the sample was vortexed vigorously. The lysate was transferred to a QIAshredder spin column and centrifuged for 2 min at 13,000 × *g*. The supernatant was transferred to new microcentrifuge tube and 0.5 volume of ethanol (96%) was added. 650 μl of the resulting sample was transferred to an RNeasy Mini spin column placed in a 2 ml collection tube and centrifuged at 8,000 × *g* for 15 s. The flow-through was discarded and 700 μl Buffer RW1 was added to the RNeasy Mini spin and centrifuged at 8,000 × *g* for 15 s, then 500 μl Buffer RPE was added and centrifuged at 8,000 × *g* for 15. This step repeated two times. Finally, total RNA was collected in 1.5 ml collection tube using 40 μl of RNase-free water and stored at −70°C before sequencing. The quantity and quality of all samples were measured on an Agilent 2100 Bioanalyzer.

### Library Construction, Transcriptome Sequencing, and Reads Alignment

The sequencing libraries were prepared at the SNP&SEQ Technology Platform at Uppsala University, Sweden. Libraries were prepared from 1 μg total RNA using the TruSeq stranded mRNA library preparation kit (Cat# 20020594/5, Illumina Inc.) including polyA selection. The library preparation was performed according to the manufacturers' protocol (#1000000040498). The libraries were sequenced on a NovaSeq SP flowcell using paired end reads and a read length of 50 bp. Raw reads were filtered for adapter sequences, low-quality sequences and ambiguous nucleotides. All downstream analyses were performed using clean data. Before aligning reads, the wheat reference genome, released by the International Wheat Genome Sequencing Consortium (IWGSC, available at (ftp://ftp.ensemblgenomes.org/pub/plants/release-43/fasta/triticum_aestivum/dna/), was downloaded and indexed using HISAT2 (https://ccb.jhu.edu/software/hisat2/). All reads then were aligned using HISAT2 and the featurCounts software (http://bioinf.wehi.edu.au/featureCounts/) was used to count the number of reads which mapped to each gene using the aligned reads and an annotation file for the wheat genome (https://plants.ensembl.org/Triticum_aestivum/Info/Index). Primary controls were performed on raw counts by filtering genes not detected across all samples. After filtering, different normalization approaches were assessed and sample qualities were evaluated by plotting the distribution of counts based on their mean variances. Correlations between samples were visualized using a variance-stabilizing-transformation (VST) to quantify the correlation between biological replicates. Moreover, the variability among samples was assessed by performing a principal component analysis (PCA) on normalized read counts.

### Differential Expression Analysis and Visualization

To assess differential expression, we used the DESeq2 package (Love et al., 2014) implemented in Rstudio (Team, 2020). For this purpose, filtered raw counts were used in a GLM model where genotype, condition and time-point were considered as covariates in the model. DESeq2 internally corrects counts for sequencing depth and RNA compositional biases using the Median of ratios method (Team, 2020). After estimating the size factor, gene-wide dispersion was performed to measure variation in the dataset. A Negative Binomial model was fitted for each gene and tests for differential expression were performed based on the values of the log-fold change using a Wald test under the null hypothesis that groups are not different and logFC = 0. Four main comparisons were made between genotypes, conditions, and time-points to identify differentially expressed genes (DEGs) at *p*-value < 0.05. To display mean expression vs log fold change for all genes in each comparison we visualized MA plots through the DESeq2 R-package. Furthermore, the EnhancedVolcano R-package (Blighe et al., 2019) was used to plot log fold change vs adjusted *p*-values. A heatmap was used to visualize clusters of DEGs based on their relative expression levels. The location of highly significant genes were plotted at their relevant chromosomes using the shinyCircos online tools (http://shinycircos.ncpgr.cn/).

### GO Enrichment, Functional Analysis, and Predication of Regulatory Elements

Gene ontology enrichment analyses were first carried out using the g-profiler database (Raudvere et al., 2019) using GO terms from wheat to determine the molecular function (MF), biological process (BP), cellular components (CC), and potential pathways. All tests were performed at a Bonferroni corrected *p*-value of 0.05. Based on the list of DEGs from each comparison, sequences of homologs rice genes were obtained using BioMart (http://plants.ensembl.org/biomart/martview/8a426f1eb50080f5f0e2770c9d719a44). These sequences were also used for GO enrichment analyses using the KOBAS software (Xie et al., 2011) to test for statistically enriched pathways in the Kyoto Encyclopedia of Genes and Genomes (KEGG, https://www.genome.jp/kegg/) database. A list of wheat gene sequences for DEGs was prepared for each comparison using the BioMart database and used as input on iTAK (Zheng et al., 2016) and PlantTFcat (Dai et al., 2013), two tools used to predict transcription factors (TFs), transcriptional regulators (TRs), and protein kinases (PKs).

### Protein-Protein Interaction (PPI) Network

To construct the PPI network we used the Search Tool for the Retrieval of Interacting Genes/Proteins database or STRING v11 (Szklarczyk et al., 2021). Before that, we extracted peptide sequences of DEGs enriched in significant biosynthetic pathways and peptide sequences of candidate transcription factors from BioMart and used a list of these sequences as the input for STRING to find putative interactions. These networks include known interactions (from curated databases and experimentally determined), predicted interactions (gene neighborhood, gene fusions, and gene co-occurrence), and other interactions (textmining, co-expression and protein homology) retrieved at high confidence interaction score of 0.7.

### Modeling 3D Protein Structure

After detecting key proteins involved in PPI networks, we used SWISS-MODEL homology modeling to predict 3D structures and quality assessments (Waterhouse et al., 2018). The SWISS-MODEL pipeline relies on ProMod3 (Studer et al., 2021) and comprises the following steps: identification of structural template(s), alignment of target sequence and template structure(s), model-building, and model quality evaluation. Through an automatic pipeline, suitable templates identifies based on BLAST (Camacho et al., 2009), and HHblits (Steinegger et al., 2019), so that the template's quality has been predicted from features of the target-template alignment. A set of 50 top-ranked templates is then selected from multiple templates according to a score obtained by combining sequence coverage and sequence similarity. Candidate models are then built based on the target-template alignment using ProMod3. To select the final models, we considered GMQE (Global Model Quality Estimation) and QMEAN (Qualitative Model Energy Analysis) scores. GMQE is a quality estimation that combines properties from the target–template alignment and the template structure. The GMQE score is expressed as a number between 0 and 1, reflecting the expected accuracy of a model built with that alignment and template, normalized by the coverage of the target sequence. Higher numbers indicate higher model reliability. On the other hand, the global and per-residue model quality were assessed through QMEAN scoring function (Studer et al., 2020).

### Validation of RNA-Seq Analysis Using RT-qPCR

To validate the result from the RNA-seq analyses, a set of nine genes were randomly selected among DEGs (Supplementary Table 2) and tested using RT-qPCR. In brief, 1.5 μg of total RNA was treated with dsDNase provided with the Thermo Scientific™ Maxima™ First Strand cDNA Synthesis Kit for RT-qPCR and used for cDNA synthesis using the following thermocycler program: incubation for 10 min at 25°C followed by 15 min at 50°C. The reaction was terminated by heating at 85°C for 5 min. The RT-qPCR reactions were performed using Thermo Scientific Luminaris HiGreen High ROX qPCR Master Mix. The reactions solution was prepared in a volume of 10 μl by using 5 μl Master Mix (2X), 0.3 μl forward and reverse primers, 1 μg cDNA and nuclease-free water up to 10 μl. A three-step cycling protocol was used with the following thermocycler program: UDG pre-treatment at 50°C for 2 min, initial denaturation at 95°C for 10 min, 40 cycles of denaturation at 92°C for 15 s, annealing at 60°C for 30 s and extension at 72°C for 30 s. To control the specificity of all products a melting curve analysis was used by increasing the temperature from 60 to 95°C and read every 0.5°C. To normalize Ct values of each reaction, an internal reference gene (*actin*) was used in the relative expression equation.

## Result

### Early Growth Under Simulated Water Deficit and Root Attributes at Tillering

**Figure 1:**
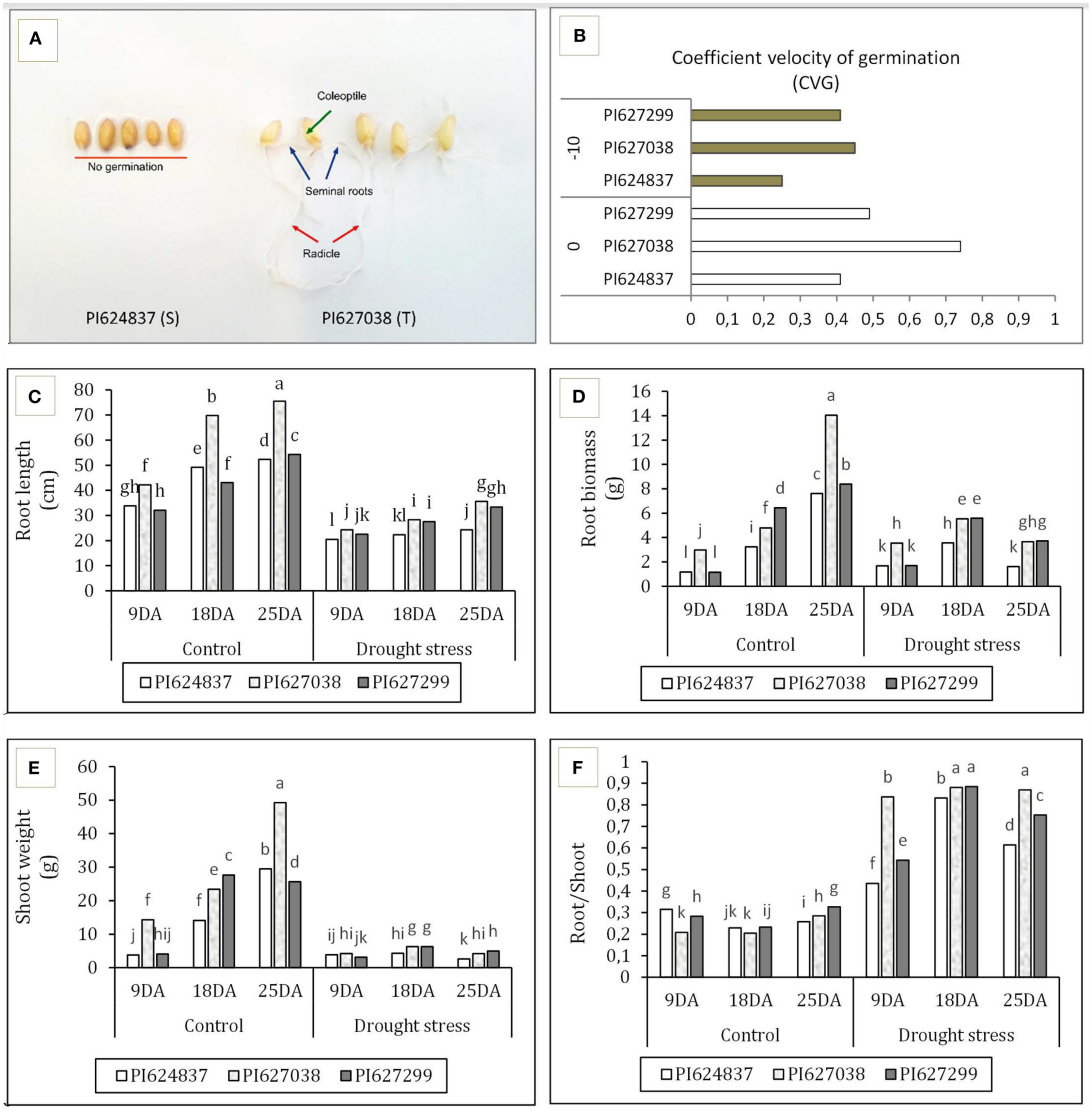
Germination study of selected wheat genotypes. **(A)** Response to simulated water deficit at−12 bar, the tolerant genotype PI627038 is showing root development including radicle, seminal roots; and shoot development including coleoptile emergence, while no germination observed in susceptible genotype PI624837. **(B)** Coefficient velocity of germination (CVG) at−10 bar. **(C)** Root length. **(D)** Root weight. **(E)** Shoot weight. **(F)** Shoot/root ratio. T, drought-tolerant; S, drought-susceptible; DA, days after applying water-deficit stress. Treatments are grouped based on Duncan multiple range test.

Water deficit reduced all germination-related traits at−10 bar, although this decrease was not significant in tolerant genotypes (Table 1). Root length under stress conditions for the two tolerant genotypes, PI627038 and PI627299, decreased ~15% relative to control conditions whereas the susceptible genotype, PI624837, suffered a reduction of ~60%. The total fresh and dry weight of seedlings was significantly reduced in all genotypes under stress conditions compared to the control. The highest total germination percentage (TGP) and normal germination percentage (NGP) was observed for PI627299 under control condition (100%). However, under simulated water deficit through PEG application, these values dropped by 90%. The susceptible genotype, PI624837, had the lowest germination percentage, both for total and for normal germination. To test the germination ability of the tolerant genotypes, a severe water stress of−12 bar was also used and the results are shown in Figure 1A. Under these conditions, the susceptible genotype PI624837, showed no germination at all. The speed of germination was calculated based on the coefficient of velocity of germination (CVG) and tolerant genotypes showed relatively high values even under a water stress of−10 bar (Figure 1B).

**Table 1:** Assessment of tolerant and susceptible genotypes according to germination parameters under simulated water deficit using PEG 6000.

**PEG (bar)**	**Genotype**	**RL (cm)**	**TFW (g)**	**TDW (g)**	**TGP (%)**	**NGP (%)**
0	PI624837 (S)	12.59 ± 1.11 ab	0.48 ± 0.06 b	0.04 ± 0.00 b	96.67 ± 4.71 ab	93.33 ± 9.43 ab
	PI627038 (T)	10.12 ± 0.76 b	0.77 ± 0.02 a	0.06 ± 0.00 ab	93.33 ± 15.28 ab	93.33 ± 10.32 ab
	PI627299 (T)	16.83 ± 3.38 a	0.88 ± 0.42 a	0.08 ± 0.01 a	100 ± 5.77 a	100 ± 5.77 a
−10	PI624837 (S)	4.61 ± 2.44 c	0.09 ± 0.10 d	0.02 ± 0.03 c	76.67 ± 5.77 c	70 ± 5.77 c
	PI627038 (T)	9.56 ± 1.62 b	0.41 ± 0.13 b	0.04 ± 0.01 b	86.67 ± 0.00 b	86.67 ± 0.00 b
	PI627299 (T)	13.63 ± 1.87 ab	0.27 ± 0.03 c	0.04 ± 0.01 b	90 ± 10.00 b	86.67 ± 11.55 b

*RL, root length; TFW, total fresh weight; TDW, total dry weight; TPG, total germination percentage; NGP, normal germination percentage; T, drought-tolerant; S, drought-susceptible. Mean values ± standard deviation, and treatments are grouped based on Duncan multiple range test. These lowercase letters are extracted from Duncan multiple range test*.

On the other hand, after a long-term application of water deficit at tillering stage, we measured root features at three time points. All four studied traits were significantly influenced by both stress treatment and sampling time. However, the studied genotypes showed different responses to control and water deficit conditions. The two tolerant genotypes had greater root length (Figure 1C), root biomass (Figure 1D), shoot weight (Figure 1E), and root/shoot ratio (Figure 1F) compared to susceptible genotype, especially under stress treatments. PI627038 (T) had the greatest root length, root and shoot biomass under normal and stress conditions at the 25 DA time point. Water deficit induced root growth and increased the root/shoot ratio for all plants although the increase for the two tolerant genotypes were greater than for the susceptible genotype.

### Biochemical Assessment of Selected Landraces

We assessed the antioxidant capacity of the three selected genotypes after applying water deficit (Supplementary Figure 1). We observed significant differences in proline, MDA, and H_2_O_2_ content and in SOD activity between the tolerant and susceptible genotypes. PI627038 and PI627299, showed the highest proline content and SOD activity under water deficit treatments (18.75 mg g^−1^ FW and 0.0683 μmol of guaiacol oxidized min^−1^ mg^−1^ protein, respectively). Water deficit increased H_2_O_2_ in all genotypes and the level of cell membrane damage increased significantly. However, damage to phospholipid membranes was greater in PI624837 (S) (46.35 μmol g^−1^ FW, MDA) compared to the tolerant genotypes.

### RNA-Seq Experiment, Gene Dispersion, and DEGs

**Figure 2:**
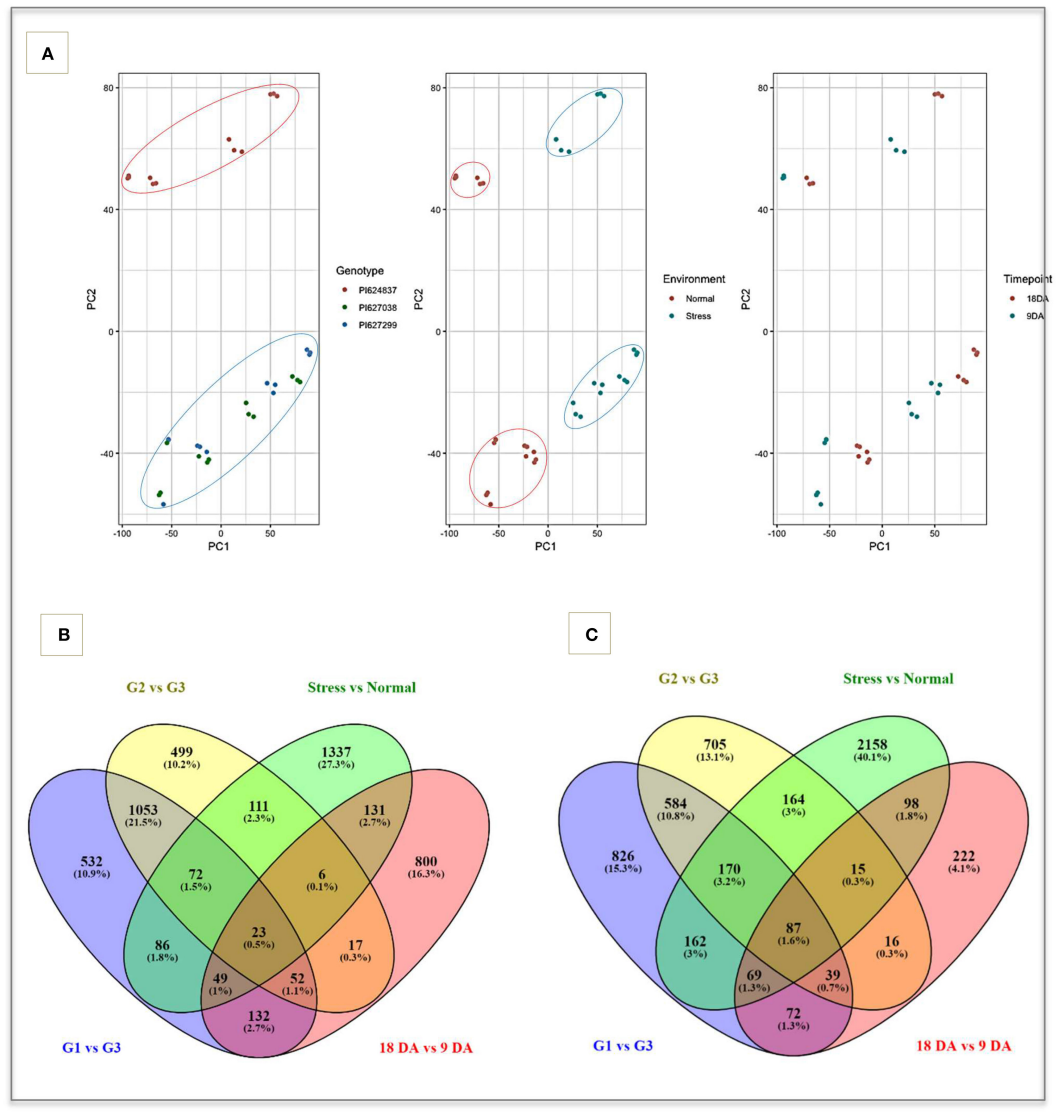
The exploited result of VST normalization method and Venn diagrams. **(A)** PCA plot of samples based on three covariates including genotypes, environments and time-points that showing differentiation between tolerant (green and blue spots) and susceptible genotypes (red spots). Venn diagram of up **(B)** and down-regulated **(C)** genes in four main comparisons. G1, PI627299 (T); G2, PI627038 (T); G3, PI624837 (S); Normal, well-watering (control); Stress, water-deficit stress; 9DA, 9 days after applying water-deficit stress; 18DA, 18 days after applying water-deficit stress; T, drought-tolerant; S, drought-susceptible.

A total of 1.06 billion clean sequencing reads were generated from the 36 cDNA libraries. The mapping rate of reads ranged from 78.1 to 84.5% across libraries without considering multi-mapped reads (average = 81.7%). The filtered and normalized data from VST were used for data exploration. The three biological replicates of each sample were highly correlated (*r* > 0.95) a consistently clustered into the same groups (Supplementary Figure 2). A principal component analysis was used to determine how variation in gene expression resulted from variation due to genotypes, conditions, and sampling time-points (Figure 2A). The PCA plots indicates that the three selected genotypes have highly differentiated expression profiles, confirming the results from field trials and other biochemical evaluations.

The gene dispersion procedure implemented in the DESeq2 package was used to estimate the variation in the dataset. This method is more reliable than using the variance and/or standard deviation of gene expression, as these measures are dependent on the mean expression level (Supplementary Figure 3). A plot of the relationship between mean counts and variance suggest that the variables are positively correlated, meaning that highly expressed genes also have higher expression variance. One way to control for this mean-variance association is to calculate the coefficient of variation which standardizes the variation by mean expression level. However, the assumption in DESeq2 is that genes with similar expression levels have similar dispersion values. Dispersion estimates are computed gene for gene using a maximum likelihood method (the internal version of dispersion in DESeq2) and gene-wise estimates are then shrunk to the fitted curve of the previously estimated dispersion. This method helps in reducing false positives in the DGEs analyses.

Putative DEGs were identified by considering a false discovery rate (FDR) of 0.05 and were further filtered to identify genes showing a FDR < 0.01 and having an absolute fold change > 2 (Figures 2B,C). In total, 14,187 unique genes were differentially expressed across the four main comparisons, with 4,738 DEGs related to the contrast between control and stress condition (1,815 up-regulated and 2,923 down-regulated), 4,008 DEGs were found when contrasting individuals PI627299 vs. PI624837 (1,999 up-regulated and 2,009 down-regulated), 3,613 DEGs for contrasting PI627038 vs. PI624837 (1,833 up-regulated and 1,780 down-regulated), and 1,828 genes when contrasting time-points 9 DA vs. 18 DA (1,210 up-regulated and 618 down-regulated). Overall, different comparisons showed some degree of overlap in both up and down-regulated genes, with the highest number of up and down-regulated overlapping genes observed between tolerant genotypes (1,053 and 584, respectively). A total of 23 up-regulated and 87 down-regulated genes were consistently expressed in all comparisons.

**Figure 3:**
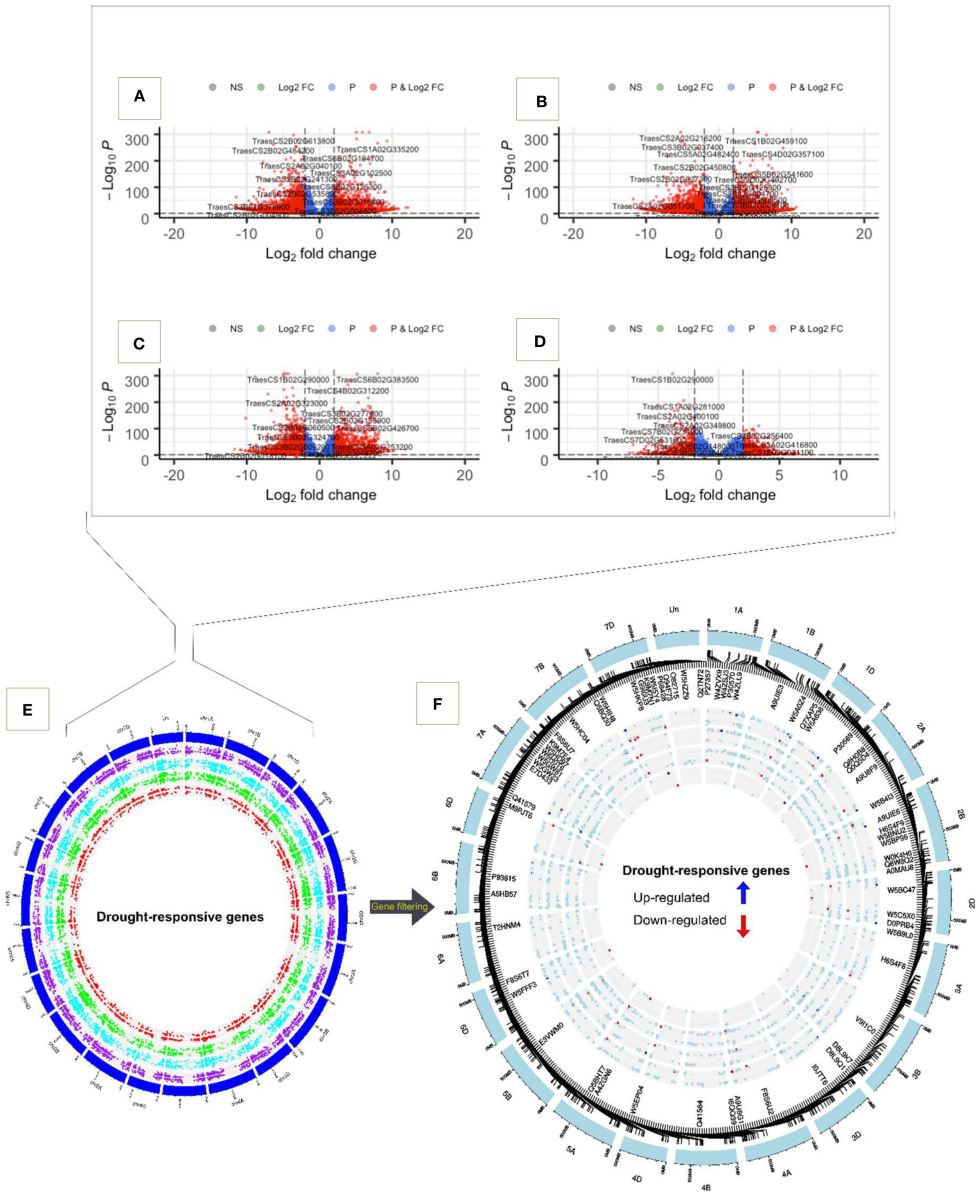
Volcano plot and chromosome wise distribution of highly significant up and down-regulated genes. **(A)** PI627299 (T) vs. PI624837 (S). **(B)** PI627038 (T) vs. PI624837 (S). **(C)** Water deficit vs. control. **(D)** Time-point 18 DA vs. 9 DA. **(E)** Circos plot of all significant genes, in comparison 1/ PI627299 (T) vs. PI624837 (S) (purple spots), in comparison 2/ PI627038 (T) vs. PI624837 (S) (light blue spots), in comparison 3/ condition water deficit vs. control (green spots), and in comparison 4/ time-point 18 DA vs. 9 DA (red spots). **(F)** Chromosomal region of important key genes and their encoding proteins are shown inside the plot.

The results of the DEG analyses were further visualized using volcano plots, in which genes are sorted by log fold changes and their adjusted *p*-values (Figure 3). In all comparisons we observed plenty of highly significant genes. For example, in the comparison between tolerant and susceptible genotypes, TraesCS1B02G459100, TraesCS4D02G360300, TraesCS4D02G357100, and TraesCS1A02G335200 were highly up-regulated while TraesCS2A02G216200, TraesCS2B02G63800, TraesCS5A02G482400, TraesCS2B02G484200, and TraesCS2B02G614100 were down-regulated (Figures 3A,B). When comparing the stress versus control condition and time-points the upregulated genes were TraesCS6B02G383500, TraesCSU02G049400, TraesCS4B02G312200, and TraesCS3B02G256400 while TraesCS1B02G290000, TraesCS7D02G201400, TraesCS5D02G046500, TraesCS1D02G280200, TraesCS1B02G290000, and TraesCS1A02G281000 were among the strongly down-regulated genes (Figures 3C,D). Visualizing the genomic location of the these genes confirmed that all chromosomes harbor genes contributing to the water deficit response (Figure 3E). A total of 1,604 genes were located on chromosomes from the B genome and 932 genes were located on chromosomes from the D genome, representing the largest and smallest number of genes when comparing genotypes PI627299 (T) and PI624837 (S). Similarly, when comparing genotypes PI627038 (T) and PI624837 (S), the B genome had the greatest proportion of genes responding to water deficit. Moreover, in the comparison between water deficit and control conditions, the D genome contained 1,611 significant genes, while the number of genes in A and B genomes were roughly equal (1,512 and 1,525 for A and B genomes, respectively). In the comparison between time-points, 639 genes responding to water deficit were located on the D genome. No drought-responsive genes located in the centromeric regions of the different chromosomes were observed. Important up and down regulated genes are distinguished in blue and red colors, respectively (Figure 3F).

A heat map of top 40 most significant genes in each comparison were plotted across all samples, showing both up and down-regulated genes (Supplementary Figure 4). Interestingly, TraesCS1A02G335200, TraesCS2B02G027300, TraesCS3B02G037400, TraesCS5B02G302500, and TraesCSB02G450800 showed different pattern of expression between the two tolerant genotypes for comparisons 1 and 2. A large fraction of the 40 selected genes showed differential expression patterns between tolerant and susceptible genotypes. With an increasing number of days after applying water stress, most genes tended to have reduced transcript levels in the different samples. The copy number of key genes are also provided in Supplementary Tables 3, 4.

### Transcription Factors Involved in Water-Deficit Tolerance

A total of 1,377 DGEs were predicted to belong to different families of transcription factors (TFs, Table 2). A set of 443 genes encoding TFs, that showed different expression patterns between normal and stress treatments, were identified. The number of TFs identified in the comparisons between tolerant and susceptible landraces were approximately the same (328 and 356 TFs, respectively). Moreover, 250 TFs were identified when the period of water deficit was increased from 9 to 18 days. Most of these TFs belong to the AP2/ERF-ERF, MYB-related, bHLH, B3, and MADS-M-type classes as genotype specific TFs. However, MYB, NAC, WRKY, C2H2, and bZIP were also major classes of TFs specific to short/long-term water deficit. Moreover, transcriptional regulators were estimated, and these largely consisted of mTERF, SNF2, TRAF, and TAZ (Supplementary Table 5).

**Table 2:** A summary of identified TFs among the different sets of DEGs in wheat.

**Comparison**	**Transcription factors**				
PI627299 vs. PI624837	AP2/ERF-ERF(19)	B3 (11)	bHLH (12)	bZIP (3)	C2C2-GATA (1)
	C2C2-CO-like(1)	C2H2 (6)	CPP (4)	GARP-G2-like (5)	GeBP (1)
	GRAS (5)	HB-other (2)	HB-HD-ZIP (5)	HSF	MADS-MIKC (1)
	MADS-M-type (6)	MYB (6)	MYB-related (17)	NAC	NF-YC (2)
	OFP (2)	PLATZ (1)	RWP-RK (1)	TCP	Tify (2)
	Trihelix (2)	TUB (2)	Whirly (3)	WRKY	zf-HD (1)
PI627038 vs. PI624837	AP2/ERF-ERF(16)	B3 (14)	bHLH (8)	bZIP (2)	C2C2-GATA (3)
	C2C2-CO-Dof (1)	C2H2 (2)	C3H	CPP (2)	CSD (2)
	E2F-DP (1)	EIL (1)	FAR1	GARP-G2-like (1)	GeBP (2)
	GRAS (8)	HB-HD-ZIP (2)	HB-other (1)	HSF (7)	LOB (1)
	MADS-MIKC (1)	MADS-M-type (10)	MYB (4)	MYB-related (11)	NAC (8)
	NF-YC (1)	PLATZ (2)	SBP (2)	TCP (1)	Tify (1)
	Trihelix (2)	WRKY (3)	Zf-HD (1)		
Water deficit vs. Control	AP2/ERF-ERF(38)	AP2/ERF-RAV (10)	B3 (6)	bHLH (21)	bZIP (12)
	C2C2-CO-Dof (1)	C2C2-Dof (5)	C2C2-GATA (1)	C2H2 (16)	C3H (1)
	ELI (1)	FAR1 (1)	GRAS (6)	HB-HD-ZIP (8)	HB-other (7)
	HB-WOX (1)	HSF (15)	LOB (2)	MADS-MIKC (2)	MADS-M-type (14)
	MYB (15)	MYB-related (23)	NAC (28)	NF-YC (4)	NF-X1 (1)
	NF-YB (1)	NF-YA (9)	OFP (1)	RWP-RK (2)	SBP
	TCP (4)	Trihelix (4)	WRKY (28)		
18 DA vs. 9 DA	AP2/ERF-ERF(45)	B3 (2)	bHLH (9)	bZIP (10)	C2C2-GATA (2)
	C2C2-CO-Dof (1)	C2C2-LSD (2)	C2C2-GATA (1)	C2H2 (8)	GARP-G2-like (5)
	GRAS (1)	HB-WOX (3)	HB-HD-ZIP (5)	HSF (6)	MADS-M-type (7)
	MYB (2)	MYB-related (9)	NAC (13)	NF-YB (2)	RWP-RK (1)
	Tify (26)	Trihelix (2)	WRKY (9)		

*The numbers in parenthesis show the actual number of the specific TFs identified in each comparison*.

### Functional Analysis Using g-Profiler

A functional analysis of DEGs with g-Profiler showed that a total of 45, 32, and 8, GO terms were involved in molecular function (MF), biological process (BP), cellular components (CC), respectively and 3 KEGG pathways were significantly enriched in the comparison between PI627299 (T) vs. PI624837 (S) (Supplementary Figure 5A). Comparing PI627038 (T) vs. PI624837 (S) revealed that 36, 27, and 6 GO terms were associated with MF, BP, and CC, respectively (Supplementary Figure 5B). In comparison between water deficit and normal conditions 92, 85, and 18 GO terms were involved in MF, BP, CC, and 5 KEGG pathways were significantly enriched (Supplementary Figure 6A). Finally, comparing time points 18 vs. 9 DA showed that 38, 32, and 9 GO terms were involved in MF, BP, CC, and 7 KEGG pathways were enriched (Supplementary Figure 6B). These findings show that extracellular region, apoplast, cell periphery, external encapsulating structure, cell wall, plasma membrane were the main cellular components affected by water deficit conditions. Furthermore, genes in box C/D snoRNP complex, ASTRA complex, intrinsic component of membrane, integral component of membrane, anchored component of membrane, extracellular space, photosystem showed significant changes in the previously mentioned cellular components.

### Functional Annotation of DEGs and KEGG Pathways on KOBAS

**Figure 4:**
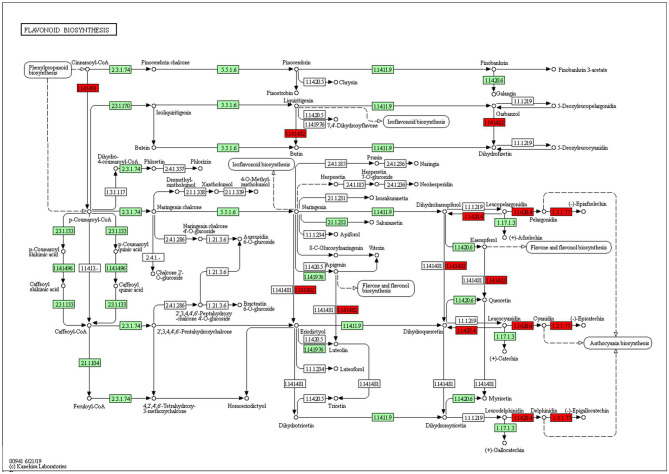
Flavonoid biosynthesis pathway. **(1.14.14.91)** Cytochrome P450 CYP73A100. **(1.14.14.82)** Flavonoid 3'-monooxygenase. **(1.14.20.4)** Leucoanthocyanidin dioxygenase. **(1.3.1.77)** Anthocyanidin reductase. Red color represents significant and characterized enzymes based on *Oryza sativa* annotation. Green color represents significant and non-characterized enzymes. White color represents characterized enzymes based on *Arabidopsis thaliana* annotation.

**Figure 5:**
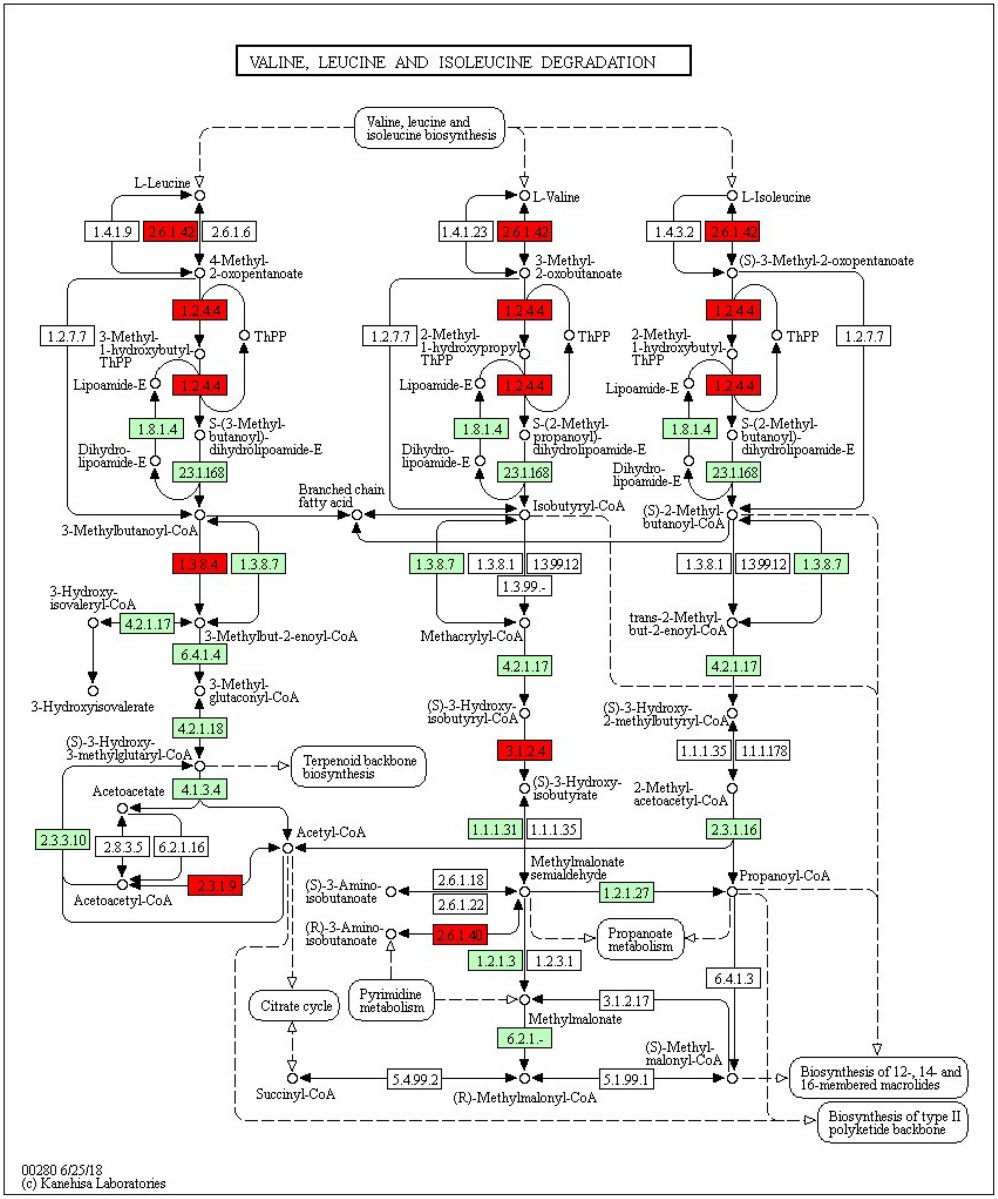
Valine, leucine and isoleucine degradation pathway. **(2.6.1.42)** Branched-chain-amino-acid aminotransferase 5, chloroplastic isoform X1. **(1.2.4.4)** 2-oxoisovalerate dehydrogenase subunit beta 1, mitochondrial. **(1.3.8.4)** Isovaleryl-CoA dehydrogenase mitochondrial. **(2.3.1.9)** Acetyl-CoA acetyltransferase, cytosolic 1. **(2.6.1.40)** Alanine–glyoxylate aminotransferase 2 homolog 1, mitochondrial. Red color represents significant and characterized enzymes based on *Oryza sativa* annotation. Green color represents significant and non-characterized enzymes. White color represents characterized enzymes based on *Arabidopsis thaliana* annotation.

**Figure 6:**
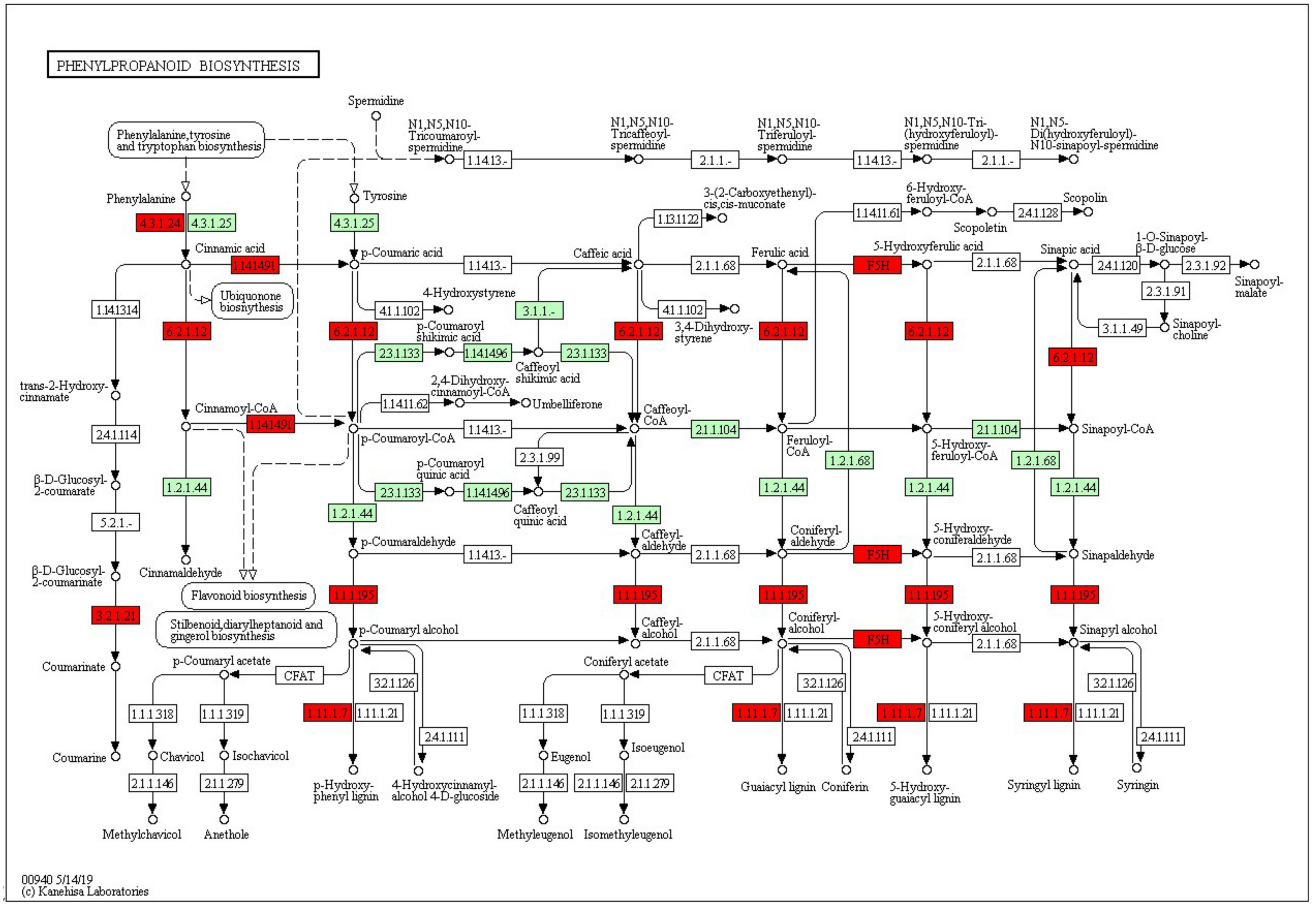
Phenylpropanoid biosynthesis pathway. **(4.3.1.24)** Phenylalanine ammonia-lyase. **(1.14.14.91)** Cytochrome P450 CYP73A100. **(F5H)** Cytochrome P450 84A1. **(3.2.1.21)** Beta-glucosidase 1. **(1.1.1.195)** Cinnamyl alcohol dehydrogenase 2. **(1.11.1.7)** Peroxidase 70-like. Red color represents significant and characterized enzymes based on *Oryza sativa* annotation. Green color represents significant and non-characterized enzymes. White color represents characterized enzymes based on *Arabidopsis thaliana* annotation.

**Figure 7:**
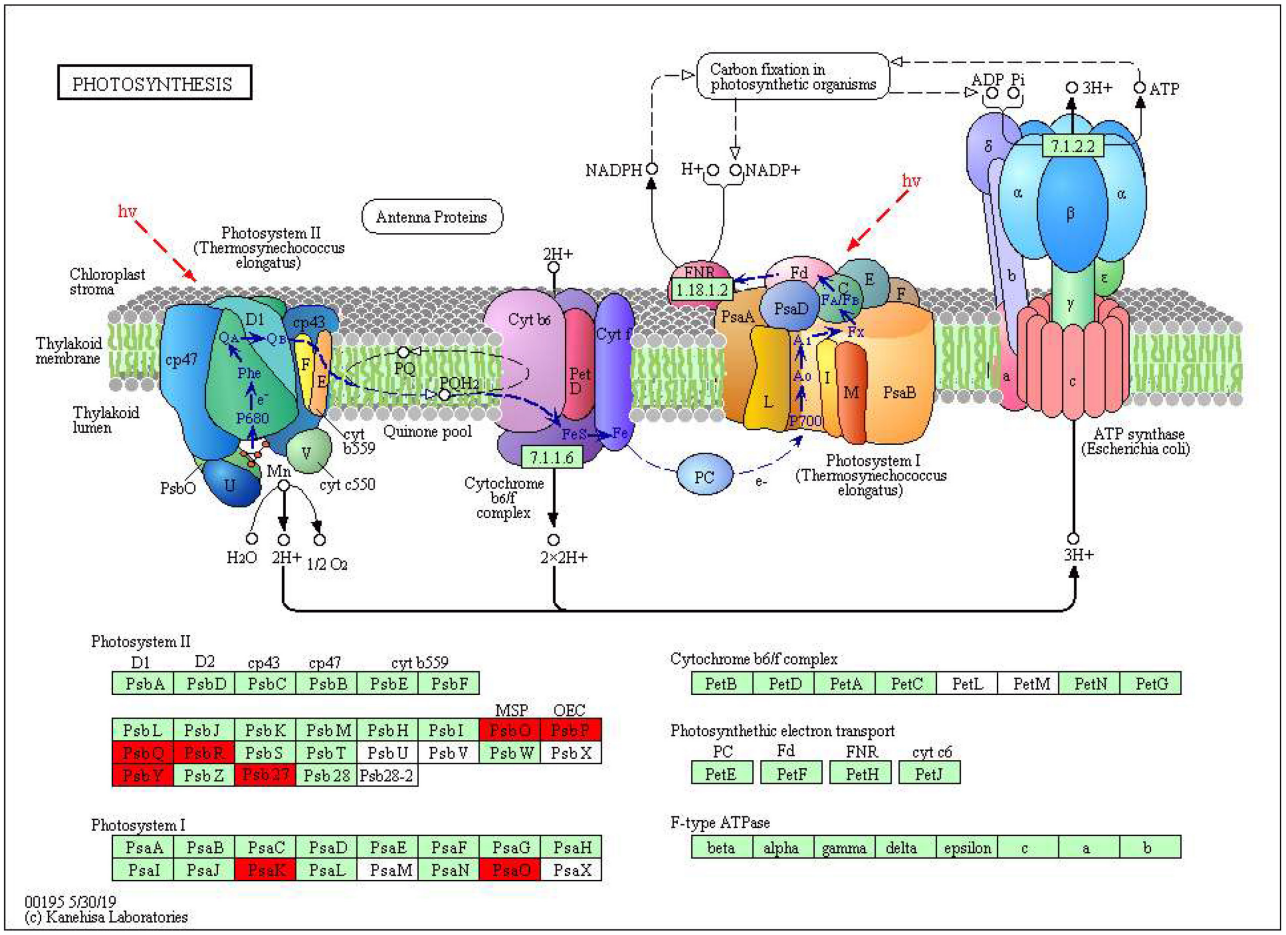
Water deficit-affected subunits of photosystems in photosynthesis chain. **(PsbO)** Oxygen-evolving enhancer protein 1. **(PsbP)** Oxygen-evolving enhancer protein 2. **(PsbQ)** Photosynthetic NDH subunit of lumenal location 2. **(PsbR)** Photosystem II 10 kDa polypeptide. **(PsbY)** Photosystem II core complex proteins psbY. **(Psb27)** Photosystem II repair protein PSB27-H1. **(PsaK)** Photosystem I reaction center subunit psaK. **(PsaO)** Photosystem I subunit O. Red color represents significant and characterized enzymes based on *Oryza sativa* annotation. Green color represents significant and non-characterized enzymes. White color represents characterized enzymes based on *Arabidopsis thaliana* annotation.

The KEGG pathways and GO terms related to DEGs were obtained from the gene enrichment analyses using KOBAS and are listed in Supplementary Tables 6–8. Five pathways, glutathione metabolism, flavonoid biosynthesis, phenylpropanoid biosynthesis, diterpenoid biosynthesis, valine, leucine, and isoleucine degradation were significantly enriched when comparing tolerant and susceptible genotypes (Supplementary Table 6). Six pathways, phenylpropanoid biosynthesis, glutathione metabolism, flavonoid biosynthesis, plant hormone signal transduction, starch and sucrose metabolism and carotenoid biosynthesis pathways were significantly enriched when comparing water deficit with normal conditions (Supplementary Table 7). Finally, five pathways, glutathione metabolism, flavonoid biosynthesis, phenylpropanoid biosynthesis, zeatin biosynthesis, and photosynthesis process, were significantly enriched between the two time points (Supplementary Table 8). These results suggest that phenylpropanoid biosynthesis, glutathione metabolism, flavonoid biosynthesis, photosynthesis process and valine, leucine and isoleucine degradation were strongly affected pathways and mechanisms in this study. In the flavonoid biosynthesis pathway, trans-cinnamate 4-monooxygenase, flavonoid 3'-monooxygenase, anthocyanidin synthase, anthocyanidin reductase were the main enzymes showing different activities between one of the tolerant landraces and the susceptible genotype (Figure 4). In the valine, leucine and isoleucine degradation process, a branched-chain amino acid aminotransferase, 2-oxoisovalerate dehydrogenase E1 component beta subunit, isovaleryl-CoA dehydrogenase, 3-hydroxyisobutyryl-CoA hydrolase, acetyl-CoA C-acetyltransferase and alanine-glyoxylate transaminase/(R)-3-amino-2-methylpropionate-pyruvate transaminase were the enzymes that showed the largest difference between the tolerant and susceptible genotype (Figure 5). In the phenylpropanoid biosynthesis pathway, the activity of phenylalanine ammonia-lyase, trans-cinnamate 4-monooxygenase, 4-coumarate–CoA ligase, ferulate-5-hydroxylase, cinnamyl-alcohol dehydrogenase, beta-glucosidase and peroxidase were considerably altered when plants were exposed to water deficit (Figure 6). Interestingly, some enriched genes identified through the functional analysis were involved in photosynthesis (Figure 7). Photosystem II 10kDa protein, photosystem II oxygen-evolving enhancer protein 1, photosystem II oxygen-evolving enhancer protein 2, photosystem II oxygen-evolving enhancer protein 3, photosystem II PsbY protein, photosystem II Psb27 protein, photosystem I subunit X and photosystem I subunit PsaO were affected after long-term water deficit. Although there are many other genes and enzymes identified for the different pathways or mechanisms, not all of them have so far been characterized, neither in rice nor in wheat. The rest of significant pathways in the different comparisons are presented in Supplementary Figures 7–12.

### PPI Network and 3D Protein Structure

To study the interaction between proteins from transcription factors families and proteins enriched in highly significant KEGG pathways, three PPIs were constructed using the STRING database. We used peptide sequences of TFs and KEGG genes as input for STRING retrieval. Genotype specific and drought-responsive interaction networks predicted 329, 212, and 259 node proteins in an enrichment *p*-value of <1.0e−16 with a high confidence interaction score of 0.7 (Table 3). This shows all three interaction networks are significantly enriched and that the proteins are expected to be biologically connected, confirming the result of the functional analysis. Networks were clustered using the specified Markov Cluster Algorithm (MCL), which is an approach for both weighted and unweighted networks. Detailed information about these three PPIs is presented in Supplementary Tables 9–11.

**Table 3:** PPI network statistics specified to tolerant genotypes and water deficit conditions.

**Variable**	**PPI1** **(PI627299 vs. PI624837)**	**PPI2** **(PI627038 vs. PI624837)**	**PPI3** **(Water deficit vs. control)**
Number of nodes	329	212	259
Number of edges	443	257	242
Average node degree	2.69	2.42	1.87
Avg. local clustering coefficient	0.138	0.183	0.0931
Expected number of edges	55	27	9
PPI enrichment *p*-value	<1.0e−16	<1.0e−16	<1.0e−16

### Genotype-Specific Protein Networks

**Figure 8:**
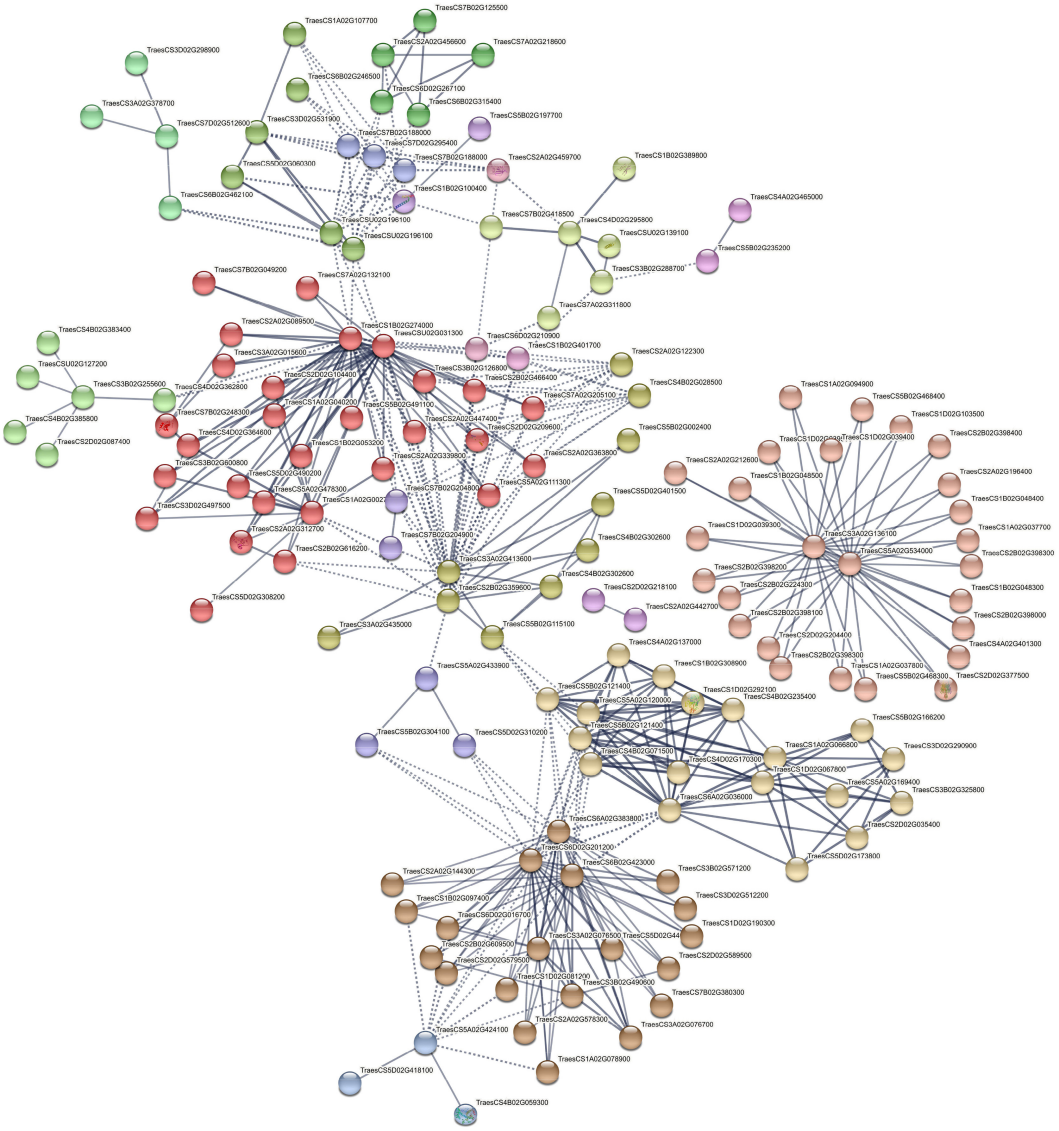
Interaction networks of related DEGs in significant KEGG pathways and putative TFs identified by comparing PI627299 (T) vs. PI624837 (S). e.g., TraesCS2D02G377500 (Phenylalanine ammonia-lyase), TraesCS5B02G11510 (MADS-box transcription factor TaAGL31), TraesCS5D02G401500 (VRN-D1), TraesCS3B02G255600 (Heat shock factor C1e), TraesCS4B02G059300 (Glutathione S-transferase 1).

Nineteen connected and disconnected clusters were obtained for PI627299 (T) vs PI624837 (S), although five clusters showed stronger interactions (Figure 8). The first cluster is showing enzymes responsible for the phenolic component's pathways (salmon-colored), and the key elements are two uncharacterized proteins belongs to the cytochrome P450 (CYP450) family located on chromosomes 3A and 5A (TraesCS3A02G136100 and TraesCS5A02G534000, respectively). These key proteins are interacting with phenylalanine ammonia-lyase (PAL, TraesCS2D02G377500) and 23 other characterized or uncharacterized proteins. The second important interaction is colored in red, and describes interactions involving glutathione S-transferase 1 (GST1, TraesCS4B02G059300, blue colored), and indicates important enzymes in glutathione metabolism. Furthermore, interactions of important transcription factors, such as MADS-box, BZIP, NAC and HsfC1e (Heat shock factor C1e,) with other proteins were also identified in several clusters.

The second enriched PPI network (PI624837 (S) vs. PI627038 (T)) showed similar interactions, while highlighting the interaction of two additional important TFs, MYB13-1 and GATA, with other proteins (Supplementary Figure 13). The key protein in this cluster was an uncharacterized protein (TraesCSU02G031300) containing a plant homeodomain (PHD). PHD is a C4HC3 zinc-finger-like motif that has been found in some nuclear proteins that are potentially involved in epigenetic and chromatin-mediated transcriptional regulation. This key protein has also a strong interaction with the MYB13-1 transcription factor, which is located at the primary shell of the network. Another important cluster colored in brown, and three key proteins including TaANS-A2 (TraesCS6A02G041800), TaANS-D1 (TraesCS6D02G004300), and an uncharacterized protein (TraesCS2B02G613000) belonging to the iron/ascorbate-dependent oxidoreductase family interacted with other 19 proteins. Both TaANS-A2 and TaANS-D1 are anthocyanidin synthase from the iron/ascorbate-dependent oxidoreductase family.

### General Protein Networks Involved in Water-Deficit Tolerance

**Figure 9:**
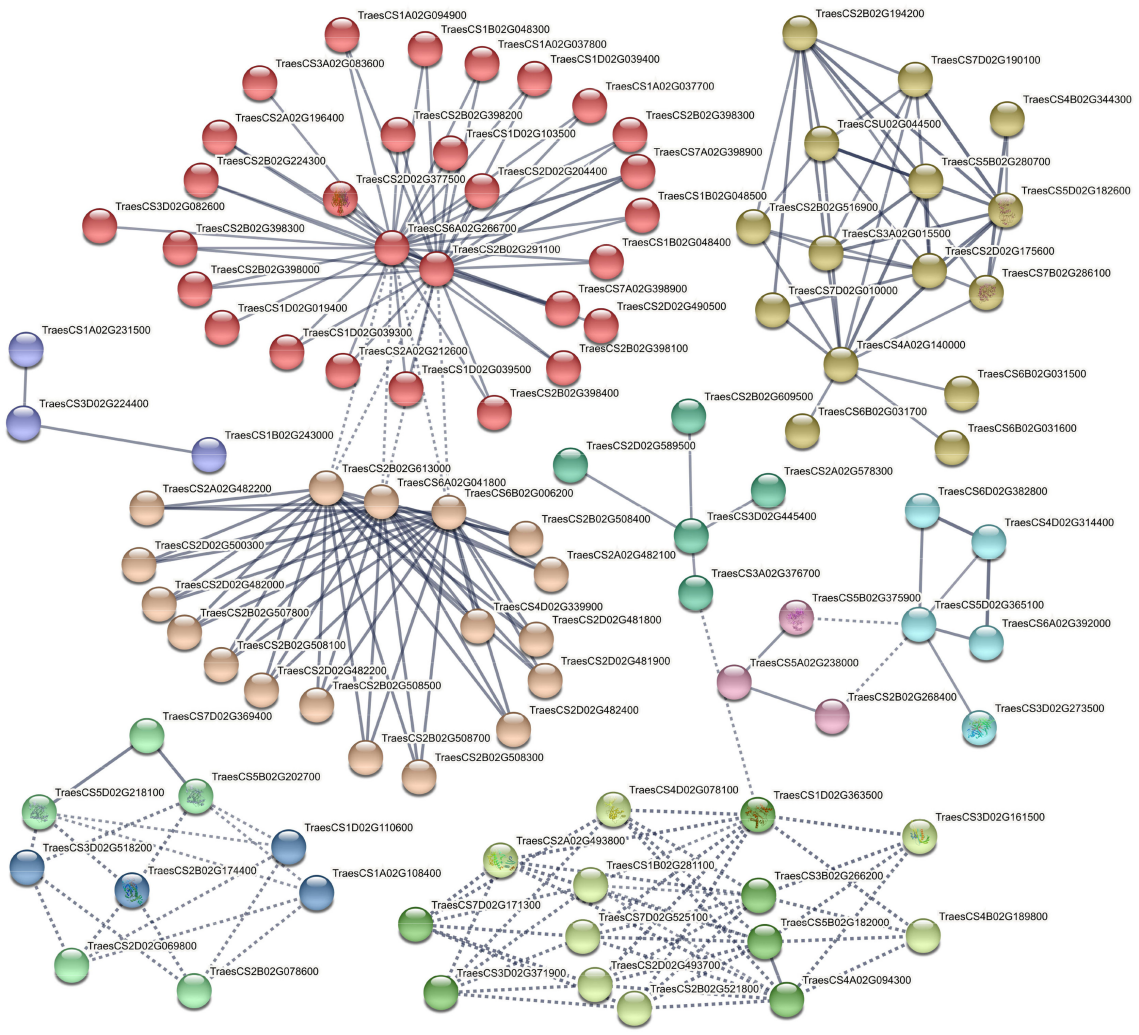
Interaction networks of related DEGs in significant KEGG pathways and putative TFs identified by comparing water deficit vs. control. e.g., TraesCS2D02G377500 (Phenylalanine ammonia-lyase), TraesCS6A02G041800 (Anthocyanidin synthase/TaANS-A2), TraesCS6A02G001500 (Anthocyanidin synthase/TaANS-D1), TraesCS7A02G539000 (Putative serine/threonine protein kinase), TraesCS2B02G174400 (1-Cys peroxiredoxin PER1), TraesCS5D02G244900 (TaABA8OH2 or ABA 8'-hydroxylase).

In the last PPI, the connection between encoded proteins from significant KEGG pathways and putative TFs obtained from water deficit vs control comparison was generated at a high confidence score and grouped into 11 important clusters (Figure 9). The first cluster contained 32 biologically connected proteins mostly with phenylalanine ammonia-lyase activity (red-colored) and cytochrome P450 family (e.g., TraesCS3A02G083600, TraesCS1D02G039300, TraesCS5B02G468300, TraesCS1D02G019400, etc.). Two uncharacterized proteins (TraesCS2B02G291100 and TraesCS6A02G266700) with AMP-binding domain were key elements and interacting with other proteins in this cluster. There was a potential connection between the first cluster and the second cluster through TaANS-A2 (TraesCS6A02G041800), TaANS-D1 (TraesCS6A02G001500), and another uncharacterized protein (TraesCS2B02G613000). In the third important cluster, the interaction between enzymes with sucrose synthase, alpha-amylase and beta-amylase activities were generated, where sucrose synthase or sucrose-cleaving enzyme provides UDP-glucose and fructose for various metabolic pathways was the key factor. However, there were also important regulatory elements in each cluster such as BZIP transcription factor (cluster 4, green-yellow), putative serine/threonine protein kinase (cluster 5, lime green), abscisic acid-inducible protein kinase (cluster 5, lime green), SNF1-type serine/threonine protein kinase (cluster 5, lime green) and ABA 8'-hydroxylase (cluster 11, pink).

### Prediction of the 3D Structure of Key Proteins

**Figure 10:**
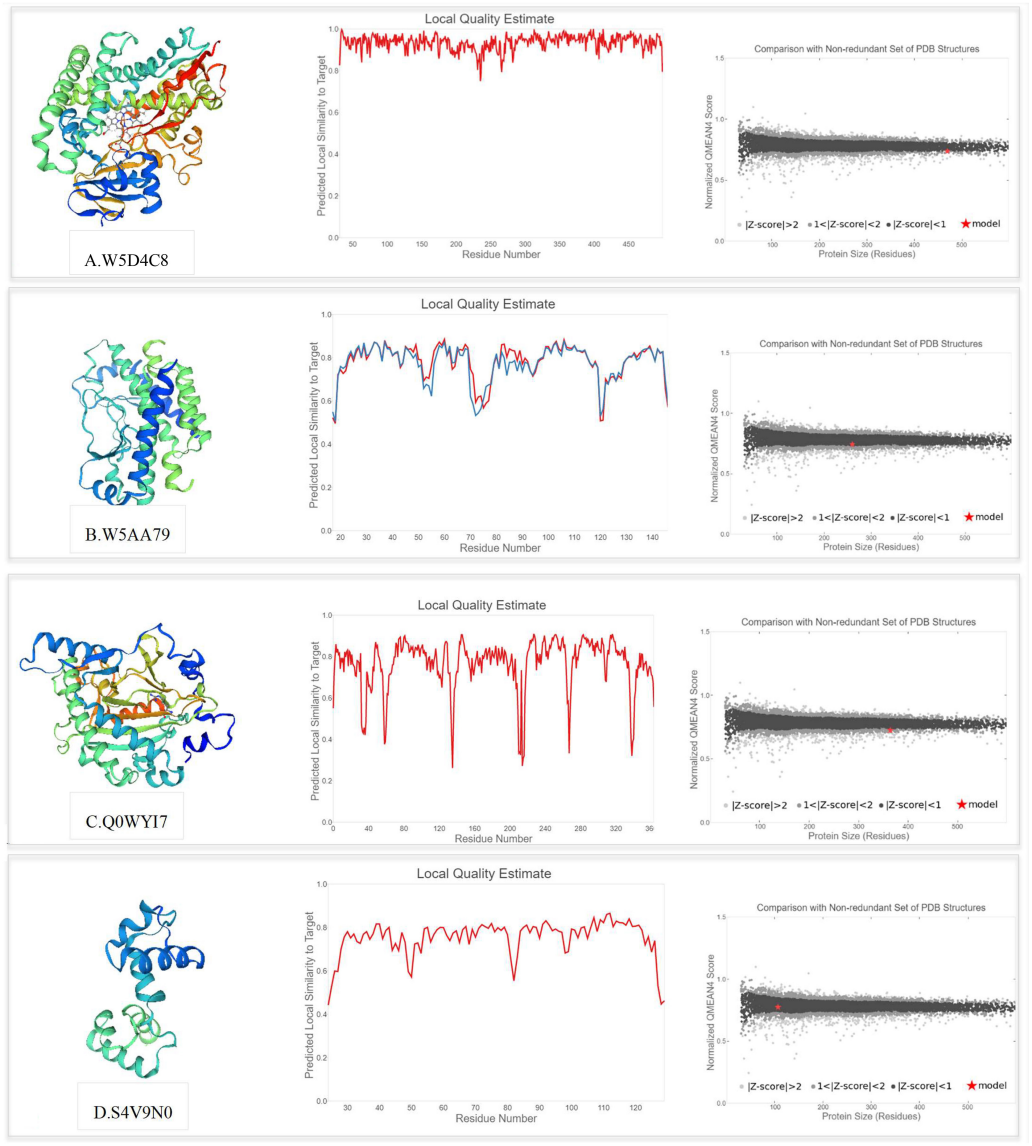
Swiss model 3D structure prediction and model accuracy based on QMEAN for following proteins (according to UniProt ID): **(A)** W5D4C8 (monooxygenase), **(B)** W5AA79 (R1 protein), **(C)** Q0WYI7 (anthocyanidin synthase), and **(D)** S4V9N0 (MYB13). The “Local Quality” plot shows, for each residue of the model (reported on the x-axis), the expected similarity to the native structure (y-axis). In the “Comparison” plot, model quality scores of individual models are related to scores obtained for experimental structures of similar size.

**Figure 11:**
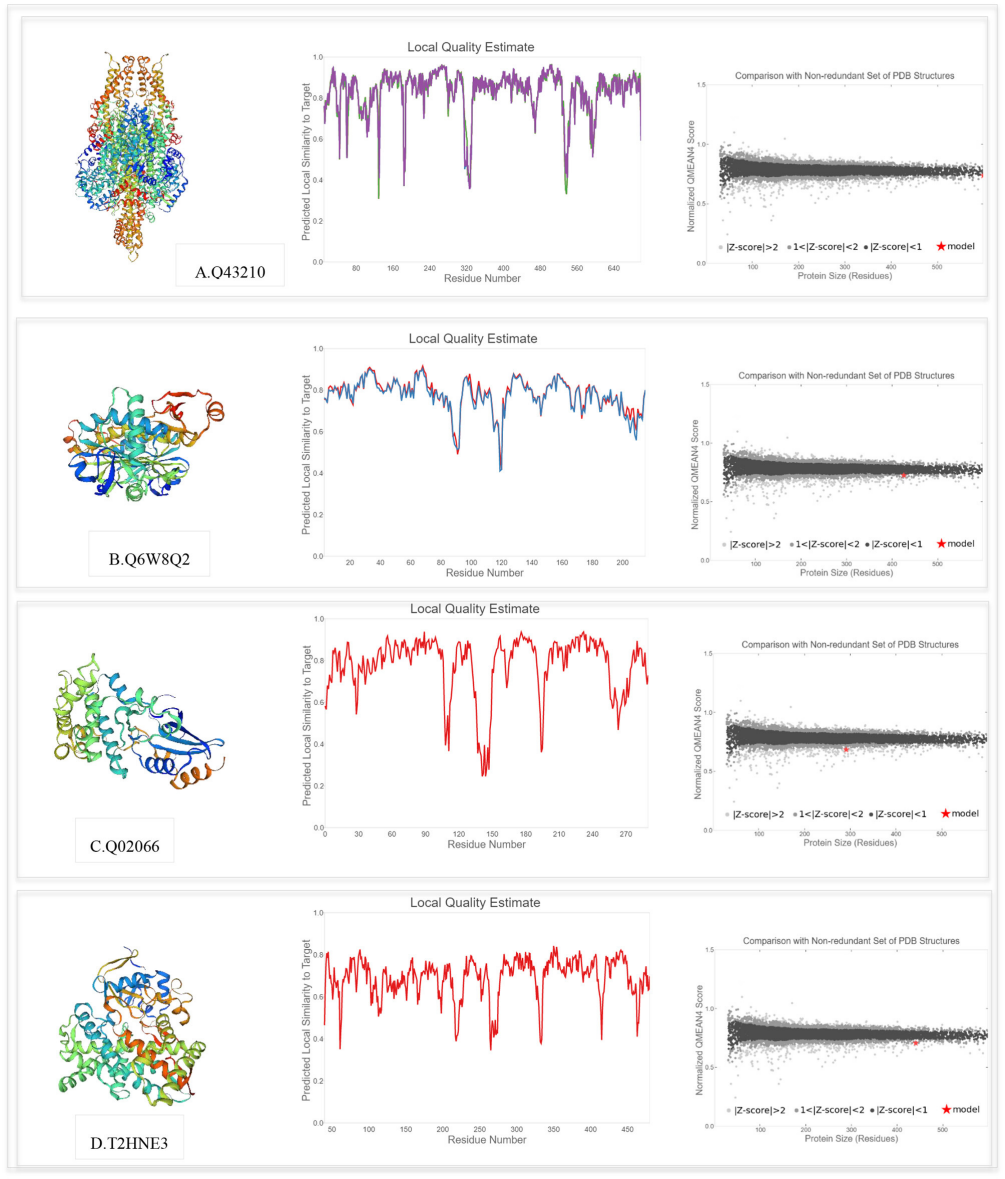
Swiss model 3D structure prediction and model accuracy based on QMEAN for following proteins (according to UniProt ID): **(A)** Q43210 (Phenylalanine ammonia-lyase), **(B)** Q6W8Q2 (1-Cys peroxiredoxin PER1), **(C)** Q02066 (Abscisic acid-inducible protein kinase), and **(D)** T2HNE3 (ABA 8'-hydroxylase*)*. The “Local Quality” plot shows, for each residue of the model (reported on the x-axis), the expected similarity to the native structure (y-axis). In the “Comparison” plot, model quality scores of individual models are related to scores obtained for experimental structures of similar size.

The SWISS-MODEL template library was searched with BLAST and HHblits for evolutionary related structures matching the target sequences of key proteins involved in PPIs. For genotype-specific networks, the 3D structures of available core proteins (according to UniProt ID) were predicted as follows (Figure 10): W5D4C8/monooxygenase (TraesCS3B02G154000), W5AA79/R1 protein (TraesCS2A02G312700), Q0WYI7/anthocyanidin synthase (TraesCS6A02G041800), and S4V9N0/MYB13 (TraesCS3A02G535100). The SWISS-MODEL template library was also searched for related structures matching the target sequences of key proteins in general drought-responsive networks (Figure 11) as follows: Q43210/phenylalanine ammonia-lyase (TraesCS2A02G381100), Q6W8Q2/1-Cys peroxiredoxin PER1 (TraesCS2B02G174400), Q02066/abscisic acid-inducible protein kinase (TraesCS2A02G493800), and T2HNE3*/*ABA 8'-hydroxylase or (+)-abscisic acid 8'-hydroxylase (TraesCS5B02G236500). Generally, higher GMQE scores observed for the predicted models in this network compared to genotype-specific networks. Templates with a similarity of more than 60% were selected to build the models. According to GMQE and QMEAN scores, predicted models were evaluated and the most reliable 3D structures with higher GMQE and QMEAN Z score close to zero were extracted for further characterization. The “Local Quality” plot shows, for each residue of the model (x-axis), the expected similarity to the native structure (y-axis). In the “Comparison” plot, model quality scores of individual models are related to scores obtained for experimental structures of similar size. The x-axis shows protein length (number of residues). The y-axis is the normalized QMEAN score. Every dot represents one experimental protein structure. Black dots are experimental structures with a normalized QMEAN score within 1 standard deviation of the mean (|*Z*-score| between 0 and 1), experimental structures with a |*Z*-score| between 1 and 2 are gray. The experimental structure that is even further from the mean is light gray. The actual model is represented as a red star.

### Experimental Validation of DEGs *via* qRT-PCR

The results of the RNA-seq analyses were validated by qRT-PCR using a total of nine randomly selected genes (five up-regulated and four down-regulated) that were normalized to the expression of the housekeeping gene *actin* (Supplementary Figure 14). The up-regulated genes were involved in rRNA N-glycosylase activity, catalytic activity, peroxidase activity, transmembrane transporter activity, ADP binding, and the down-regulated genes were involved in carbohydrate binding, nucleic acid binding, inorganic phosphate transmembrane transporter activity, methyltransferase activity, respectively. The expression pattern (up and down-regulations) observed in the qRT-PCR experiment was highly similar to the observed RNA-seq log2 fold change for all selected genes (RSquare and adjusted RSquare were 0.90 and 0.89, respectively). This shows that the results from the transcriptomics survey are highly reliable.

## Discussion

Plant productivity is mainly affected by water deficit during different developmental stages, especially in arid and semi-arid areas. In the current study, we have compared drought tolerant and susceptible wheat genotypes at germination (Zadoks 05–09) and tillering (Zadoks 20–25) stages in terms of important root attributes and antioxidant capacity. We have further evaluated whole root transcriptomes to elucidate specific and general genes and protein pathways that contribute to water-deficit tolerance in plants, and which are interesting targets for molecular breeding programs or for manipulation with genetic engineering approaches.

### Root Development and Radical Scavenging Activity of Contrasting Genotypes

Studying wheat landraces at an early stage using PEG 6000 at−10 bar showed a reduction in germination percentage, root length, and root biomass in both tolerant and susceptible genotypes, although tolerant genotypes showed smaller reductions in all traits. Tolerant genotypes were able to easily germinate even under severe water stress conditions (−10 and−12 bar) and showed higher germination speeds. Our study at tillering stage, showed that water deficit is followed by the accumulation of reactive oxygen species (ROS), such as H_2_O_2_, in leaves. H_2_O_2_ then further induce lipid peroxidation in phospholipid membranes yielding MDA (Czarnocka and Karpiński, 2018). MDA is the result of peroxidation of poly-unsaturated fatty acids which seems to be a signaling mechanism in response to stresses (De Dios Alché, 2019). In this study, the concentration of both H_2_O_2_ and MDA in PI627038 (T) and PI627299 (T) was steadily lower than PI624837 (S). After the production of ROS, plants deploy radical scavenging systems, either through enzymatic or non-enzymatic mechanisms. It has been shown, antioxidant enzymes, such as SOD, could function in radical scavenging efficiently enough to reduce oxidative damage (Ashraf, 2009; Caverzan et al., 2016; Kapoor et al., 2019). In our study, SOD showed higher activity after water deficit, although greater activities were observed in tolerant genotypes.

Another important plant response to stress is the accumulation of a range of metabolites e.g., amino acids. Proline is an amino acid that plays a crucial role in plant metabolism, especially under stressful conditions, as it contributes to antioxidant defense, signaling, metal chelating and most importantly acts as an osmolyte (Hayat et al., 2012; Kahraman et al., 2019). Our findings suggest that also proline content increase under stress treatment, compared to control conditions. We observed significant differences in the accumulation of proline between tolerant and susceptible genotypes under stress conditions. The overproduction of proline under stressful conditions would further convey stress tolerance through maintaining osmotic and ROS balance in cells, and ultimately prevents electrolyte leakage and oxidative damage in plants (Hayat et al., 2012; Liang et al., 2013; Forlani et al., 2019).

Previous studies suggest that plants usually extend lateral and embryo-formed roots when encountering drought stress conditions (Kashiwagi et al., 2005; Moumeni et al., 2011; Ahmadi et al., 2018; Bristiel et al., 2019). In the present study, tolerant genotypes showed deeper roots and more root biomass under both control and water deficit conditions and especially after 18 and 25 days of stress treatment. The total fresh biomass of roots and shoots generally declined during the water stress experiment, indicating the negative effect of water deficiency on the development of roots and shoots in wheat. The root/shoot ratio tends to increase under stress conditions, suggesting that plants invest more assimilates into developing the length and density of roots by extending lateral and embryo roots. However, the pilot investigation showed that more root biomass in tolerant genotypes was due to an increase in the number of lateral and adventitious root, rather than a production of deeper roots. In addition to environmental factors, root development mainly relies on hormone homeostasis, including auxin and abscisic acid (ABA) content. Auxin contributes to cell elongation by modulating the H^+^ secretion through adjusting PM H^+^-ATPase enzyme activity (Rober-Kleber et al., 2003; Fleming, 2006; Staal et al., 2011; Yuan et al., 2017; Ogura et al., 2019). Furthermore, ABA will accumulates in root tips after sensing drought and is crucial for maintaining root elongation (Sengupta et al., 2011; Xu et al., 2013). ABA functions in cell wall extensibility and regulate indole-3-acetic acid (IAA) transport, which further enhance root elongation and the generation of hairy roots (Yamaguchi and Sharp, 2010; Xu et al., 2013; Ma et al., 2019; Rosales et al., 2019).

### Differential Gene Expression and Pathway Enrichment

In addition to understanding of hormone homeostasis, dissecting the expression pattern of other biological pathways involved in root development and in plant response to water deficit will further provide fundamental information for manipulating and exploiting these biological processes in order to improve the adaptability of plants. Our initial hypothesis was that differences in root morphology and antioxidant capacity between drought tolerant and susceptible genotypes is correlated with the differential expression of key genes, including transcription factors, and stress response pathways. We therefore used RNA-seq to enable us to detect DEGs and then used functional enrichment derived from these genes to build and characterize putative gene and protein interaction networks that contribute to root development. DEGs were extracted by pairwise comparisons between wheat genotypes, different drought conditions and time-points during the drought period. These analyses illuminate genetic differences between genotypes as well as responsive genes to drought. Finally, this should enable us to identify genes that are consistently drought-responsive under long-term water shortage in wheat.

We observed distinct differences in the transcriptome comparisons. A total of 14,187 genes were identified as being differentially expressed across comparisons, highlighting genes that potentially contribute to drought tolerance through up or down regulations. Some genes overlapped in both comparisons while others were unique to different tolerant genotypes. The number of down-regulated genes were generally higher than the number of up-regulated genes. Interestingly, we observed a significantly higher number of genotype-specific DEGs in the susceptible genotype under stress and non-stress conditions compared to the tolerant genotypes, in line with earlier observations by Fracasso et al. (2016) and Mia et al. (2020) who also reported similar expression pattern changes in susceptible materials. The DEGs that were down-regulated showed GO enrichment associated to catalytic activities, oxidative reactions, ion binding, protein kinase activity and antioxidant activity under both short-term and long-term water deficit. In contrast, up-regulated genes in the susceptible genotype under short and long-term water deficit were associated with transmembrane transporter activity, transporter activity and response to water processes. These findings suggest a lower focus on drought tolerance mechanisms in susceptible plants and that they invest more assimilate into the biosynthesis of secondary metabolites and metabolic pathways, resulting in higher energy consumption and consequently lower adaptation to drought (Mia et al., 2020).

Down-regulated genes under short-term water deficit in tolerant plants were significantly associated with nicotianamine synthase activity, tricarboxylic acid biosynthetic process and cellular biogenic amine biosynthetic process while up-regulated genes were mainly linked to peroxidase activity, oxidoreductase activity, antioxidant activity, heme binding, hydrogen peroxide catabolic process, response to oxidative stress, response to water and reactive oxygen species metabolic process. Interestingly, by increasing the time of water deficit from 9 to 18 days, we observed a downregulation in catalytic activity, oxidoreductase activity, heme binding, peroxidase activity, and antioxidant activity also in tolerant genotypes. At the same ime, up-regulated DEGs in tolerant genotypes were associated with phosphatase activity, protein serine/threonine phosphatase activity, phosphoric ester hydrolase activity, chitin-binding, DNA-binding transcription factor activity and sequence-specific DNA binding. These results suggest the existence of a water-deficit response threshold in these plants. In other words, tolerant genotypes would up-regulate genes and activate more pathways connected to water-deficit tolerance upon required and then they will active other regulatory gene networks involved in processes rather than tolerance. A further analysis of up-regulated genes showed that the most common responsive pathways to water deficit were the flavonoid pathway (pathway ID ko 00941), glutathione metabolism (ko 00480), phenylpropanoid biosynthesis (ko 00940), and diterpenoid biosynthesis (ko 00904). Accumulation of secondary metabolites such as flavonoids and phenylpropanoids occur when plants are exposed to drought and other stresses (Sharma et al., 2019). These products are part of the antioxidant defense system and scavenge reactive oxygen species (ROS) that are induced upon oxidative stress (Ma et al., 2014; Tattini et al., 2015; Niinemets, 2016). Phenylpropanoids are among the largest group of plant secondary metabolites and are formed from aromatic amino acids like phenylalanine and tyrosine. The final products in this pathway are flavonoids, phenolic acids, monolignols, coumarins, and stilbenes. Crucial regulatory enzymes, such as phenylalanine ammonia lyase (PAL), chalcone synthase (CHS), and transcription factors belonging to the MYB family, WD40-type or basic helix-loop-helix (bHLH) classes are well-known members of the phenylpropanoids pathway and could therefore be keye targets in genetic engineering experiments aimed at increasing wheat adaptation to drought (Falcone Ferreyra et al., 2012; Sharma et al., 2020; Verma et al., 2020). In our study, 33 genes encoding enzymes such as phenylalanine ammonia-lyase (K10775), cinnamoyl-CoA reductase 1 (K09753), peroxidase 70-like (K00430), and beta-glucosidase 1 (K05350) were up-regulated in the phenylpropanoid pathway. Seven genes encoding enzymes, such as ent-copalyl diphosphate synthase 1 (K04120), ent-kaurene oxidase 2 isoform X3 (K04122), ent-kaurene oxidase-like 5 (K21719), and 9-beta-pimara-7,15-diene oxidase (K16085) were upregulated in the diterpenoid pathway. In a previous study on roots of two pairs of drought-tolerant and susceptible rice near-isogenic lines, Moumeni et al. (2011) showed that a set of highly up regulated genes were mostly involved in secondary metabolism, response to stimulus, amino acid metabolism and signal transduction, while down-regulated genes were mainly responsible in photosynthesis and cell wall growth.

### Predication of Regulatory Elements

Further analyses of regulatory factors highlighted the role of, for example, AP2/ERF-ERF, MYB-related, bHLH, B3, MADS-M-type, MYB, NAC, and WRKY as specific TFs affecting downstream genes and biosynthesis pathways. However, AP2/ERF-ERF, MYB-related, bHLH, and B3 transcription factor families were genotype-specific regulatory elements under water-deficit treatments in the present study. Transcription factors or *trans*-acting elements are the core of regulatory networks of downstream genes through binding to *cis*-acting elements in the promoters of target genes and thereby play a crucial role in modulating gene expression (Yamaguchi-Shinozaki and Shinozaki, 2006; Todaka et al., 2012; Nakashima et al., 2014; Xie et al., 2019). Among the TFs, the APETALA 2/ethylene-responsive element binding factor (AP2/ERF) which contain a conserved DNA-binding domain is well-known to harbor a large group of TFs in plants (Sakuma et al., 2002; Xie et al., 2019). This family includes four main subfamilies: the AP2, ERF, RAV, and DREB (dehydration-responsive element-binding protein), though the DREB subfamily is more well-characterized and family members are known to be involved in response to abiotic stress responses and especially drought stress (Yamaguchi-Shinozaki and Shinozaki, 2006; Mizoi et al., 2012; Xie et al., 2019). However, this response occurs *via* binding of these TFs to cis-acting dehydration-responsive element/C-repeat (DRE/CRT) domain in the promoters of target genes (Sakuma et al., 2002; Mizoi et al., 2012). Our results show that 19 and 16 TFs belonging to the AP2/ERF family members were involved in response to water deficit in tolerant genotypes.

The second important TF group we identified was the MYB-related subfamily with a single SANT domain (17 and 11 members in tolerant genotypes, respectively). Generally, MYB family genes can be classified into four subfamilies according to the number of repeats in the sequence (ranging from 1 to 4) including one repeat/MYB-related (1R-MYB), two repeats (R2R3-MYB), three repeats (3R-MYB), and four repeats or 4R-MYB (Ambawat et al., 2013; Xiong et al., 2014; Baldoni et al., 2015). Although it has been suggested R2R3-MYB type are primarily involved in response to various abiotic stresses (Xiong et al., 2014), our findings suggest that 1R-MYB could also contribute to drought-responsive regulatory networks. In line with other TFs, MYB proteins have two unique regions, a conserved N-terminal (DNA-binding domain) and a diverse C-terminal that determines the regulatory potential of the protein (Ambawat et al., 2013). Previous research have shown these TFs are mostly involved in the ABA-signaling pathway, phenylpropanoid metabolism, auxin signaling, secondary metabolite pathways and interestingly the activation of lateral root meristem under water deficit conditions (Seo and Park, 2009; Ambawat et al., 2013; Baldoni et al., 2015; Zhang et al., 2020).

Another important genotype-specific TF family we detected in the present study was *bHLH* or basic helix-loop-helix protein, which are known to contribute to a wide range of process in plants, such as plant metabolism, light signal transduction, photomorphogenesis and, more importantly, response to various stresses (Mao et al., 2017; Sun et al., 2018). This TF family contains a highly conserved basic/helix-loop-helix domain, which is formed by two different sections located at the N-terminal and C-terminal of the bHLH (Sun et al., 2018). The first part is a basic amino acid region (at N-terminus) and the second part is the helix-loop-helix region/HLH (at C-terminus). These TFs identify and bind to the target sequences using the basic amino acid region, while the HLH domain provides domain dimerization and further triggers protein-protein interactions (Niu et al., 2017; Tanabe et al., 2019). This in turn lead to the establishment of homodimeric or heterodimeric complexes which eventually regulates gene transcription (Sun et al., 2018; Tanabe et al., 2019). Previous studies suggest that members of this transcription factor family improve drought tolerance *via* inducing genes involved in ABA signaling, osmotic capacity, ROS scavenging, flavonoids pathway genes and launching other signaling cascades (Dong et al., 2014; Ji et al., 2016; Wang et al., 2016). Our results identified 12 and 8 TFs from the bHLH family that are involved in the drought response cascade in tolerant genotypes. Janiak et al. (2019) showed 13 transcription factors from DREB, WRKYs, AP2, NACs, MYB or MYB-related, bHLH, and bZIP TFs were the main TFs families affected under mild drought stress in barely as well.

### Protein-Protein Interactions

The prediction of probable interactions between DEG-encoded proteins and transcription factors through co-expression, co-occurrence, fusions networks or other analyses provides a functionally characterized framework for further studies. Our results suggest that PPI networks in tolerant wheat landraces were relatively similar, although some unique differences were also observed. In both genotypes, the interactions between enzymes involved in phenolic component pathways, antioxidant defense system and glutathione metabolism were the main source of similarity. Two important proteins belonging to the CYP450 family were connected to PAL isozymes in the first cluster of PPI1 (Figure 8). Cytochromes P450 (CYPs) members form one of the largest and most important enzymatic protein families in plants (around 1% of total gene annotations in plant species) and they are involved in various processes, such as developmental, physiological, signaling, and defense systems in plants (Mizutani and Ohta, 2010; Wei and Chen, 2018; Magwanga et al., 2019). The crucial role of CYPs in primary and secondary metabolism occurs by catalyzing a broad range of monooxygenation/hydroxylation reactions (Pandian et al., 2020). The catalytic function of CYP73, CYP98, CYP84, CYP96B4, CYP709B1, CYP709B2, and CYP709B3 in the phenylpropanoid pathway, that provide structural phenolic compounds e.g., lignin and suberin, antioxidants components like polyphenols, and other protectant compounds such as flavonoids, has previously been reported for several plant species (Mizutani and Ohta, 2010; Mao et al., 2013; Wei and Chen, 2018). Duan et al. (2017) showed overexpression of *SoCYP85A1* (Spinach Cytochrome p450) gene in transgenic tobacco could increase primary root length and lateral roots that resulted in enhanced drought tolerance of transgenic plants with respect to wild type. More recently, overexpression of *CsCYT75B1* (Citrus Cytochrome P450) gene in transgenic Arabidopsis significantly increased the total contents of flavonoid and ultimate antioxidant capacity (Rao et al., 2020). In addition to mentioned CYP450 proteins in PPI1, another three CYP450 showed interactions with proteins belong to the iron/ascorbate-dependent oxidoreductase family in PPI2. In PPI3, we observed other isozymes of CYP450 interacting with beta-carotene hydroxylase A1, beta-carotene hydroxylase D1, and proteins in the carotenoid pathway. TaABA8OH2 or ABA 8'-hydroxylase belongs to the CYP707 family and catalyzes the conversion of ABA to 8'-hydroxy ABA and then phaseic acid (PA) through isomerization under drought conditions (Pandian et al., 2020). A dynamic balance between the biosynthesis and catabolism of ABA is interceded *via* ABA 8'-hydroxylase and determines changes in downstream processes controlled by ABA (Zheng et al., 2012; Pandian et al., 2020).

In the present study, GST enzymes were also significantly enriched for interacted with other proteins, in which GSTA1 showed a concrete relationship with a 219 amino acid (aa) protein as a mediator of other GSTs. These enzymes contribute to many functions related to stress tolerance through the detoxification of endobiotic and xenobiotic compounds. This occurs *via* conjugating reduced glutathione (tripeptide y-Glu-Cys-Gly) to hydrophobic electrophiles (Ji et al., 2010; Rezaei et al., 2013; Islam et al., 2019). On the other hand, drought-responsive transcription factors and protein kinases were significantly enriched in all networks. For TFs, MYB13-1, MADS box, GATA, bZIP, GRAS family, NAC2B, and the GATA transcription factors all demonstrated crucial interactions. The putative serine/threonine protein kinase (PK4), abscisic acid-inducible protein kinase, and SNF1-type serine-threonine protein kinase also showed important connections in several clusters.

## Conclusion

We report putative mechanisms and biosynthetic pathways that are responsive in wheat roots in response to water-deficit stress. Investigation of contrasting drought tolerant and susceptible wheat genotypes allowed us to elucidate transcriptome changes, dynamic regulatory networks, and protein-protein interactions that are important under adverse conditions. In total, more than 14,000 genes were differentially expressed (DEG) and most of these genes mapped to the B genome of wheat. A significant proportion of these DEGs were enriched in flavonoid biosynthesis, phenylpropanoid biosynthesis, glutathione metabolism, diterpenoid biosynthesis, and hormone signaling pathways. Further, inspection of the DEGs identified 1,377 DEGs that potentially function as transcription factors and moderate downstream cascades in response to water deficit. These TFs were dominantly from AP2/ERF-ERF, MYB-related, bHLH, WRKY, and NAC families. We also modeled three protein-protein interaction networks (PPIs), utilizing significant KEGG proteins and putative TFs to predict and characterize interaction networks. It appears that several enzymes from the CYP450, TaABA8OH2, PAL, and GST families play conclusive roles in water-deficit tolerance in connection with MYB13-1, MADS-box, and NAC transcription factors and the protein kinase superfamily.

## Data Availability Statement

The datasets presented in this study can be found in online repositories. The names of the repository/repositories and accession number(s) can be found at: ENA under the accession number PRJEB44859 (https://www.ebi.ac.uk/ena/browser/view/PRJEB44859).

## Author Contributions

YR: designed and performed the experiments, analyzed the datasets, and wrote the manuscript. PI: assisted in designing the RNA-seq experiment and root studies panel, transcriptome analysis, and writing the manuscript. MB: assisted in biochemical and germination studies and edited the manuscript. HA: assisted in designing the RNA-seq experiment and edited the manuscript. AT: edited the manuscript. SK: assisted in biochemical and germination studies. All authors contributed to the article and approved the submitted version.

## Conflict of Interest

The authors declare that the research was conducted in the absence of any commercial or financial relationships that could be construed as a potential conflict of interest.

## Publisher's Note

All claims expressed in this article are solely those of the authors and do not necessarily represent those of their affiliated organizations, or those of the publisher, the editors and the reviewers. Any product that may be evaluated in this article, or claim that may be made by its manufacturer, is not guaranteed or endorsed by the publisher.
