# Supplementary material for: Characterization of Dynamic Regulatory Gene and Protein Networks in Wheat Roots Upon Perceiving Water Deficit Through Comparative Transcriptomics Survey
Source: Front Plant Sci. 2021 Aug 16;12:710867. doi: 10.3389/fpls.2021.710867 (PMC8415571; doi:10.3389/fpls.2021.710867)
Supplement: Supplementary file 1 [file Data_Sheet_1.zip › Supplementary Figures S1-S14.DOCX]

**Figure S1.** Antioxidant and osmoprotectant capacity of tolerant and susceptible wheat lines. **(A)** proline content. **(B)** malondialdehyde content. **(C)** hydrogen peroxide concentration. **(D)** superoxide dismutase activity.


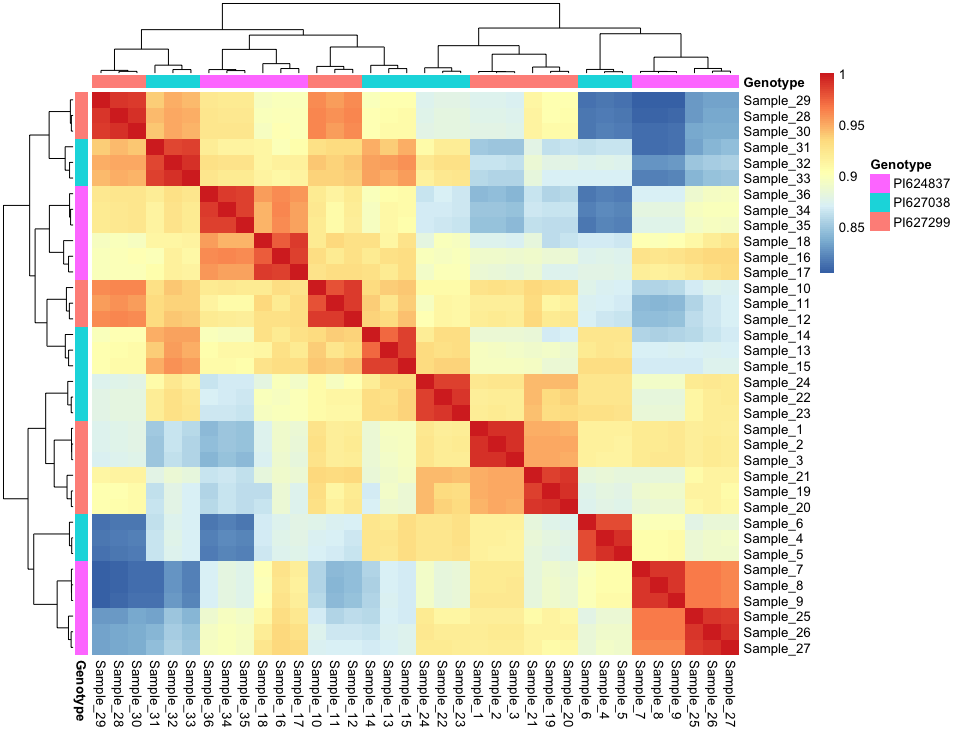


**Figure S2.** The exploited result of VST normalization method, correlation between biological replicates of samples through heatmap plot.


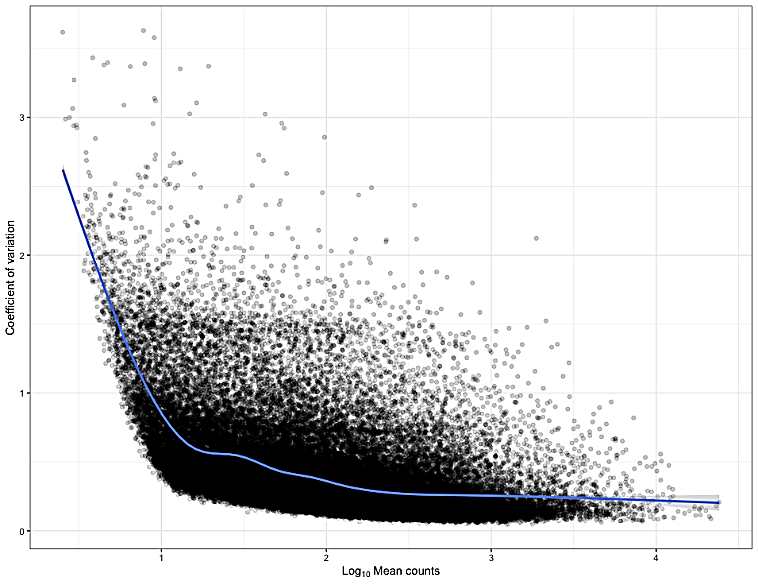

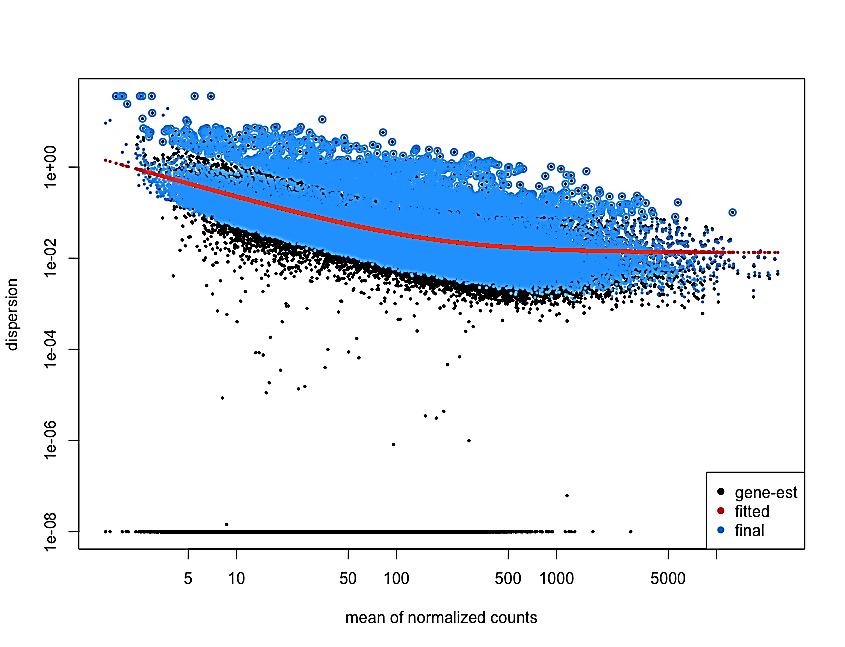


A

B

**Figure S3.** The result of gene dispersion procedure in the DESeq2 package. **(A)** Plotting mean counts against coefficient of variation. **(B)** DESeq2 gene dispersion procedure. Black points represent the maximum likelihood dispersion estimate for each gene, blue points represents the new gene dispersion estimate after they are shrunk towards the fitted curve (shown in red).

A


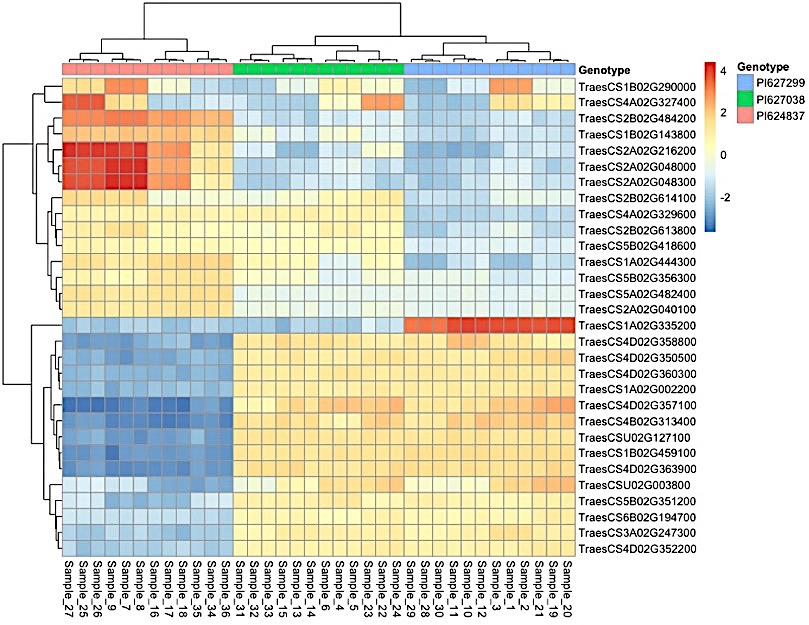

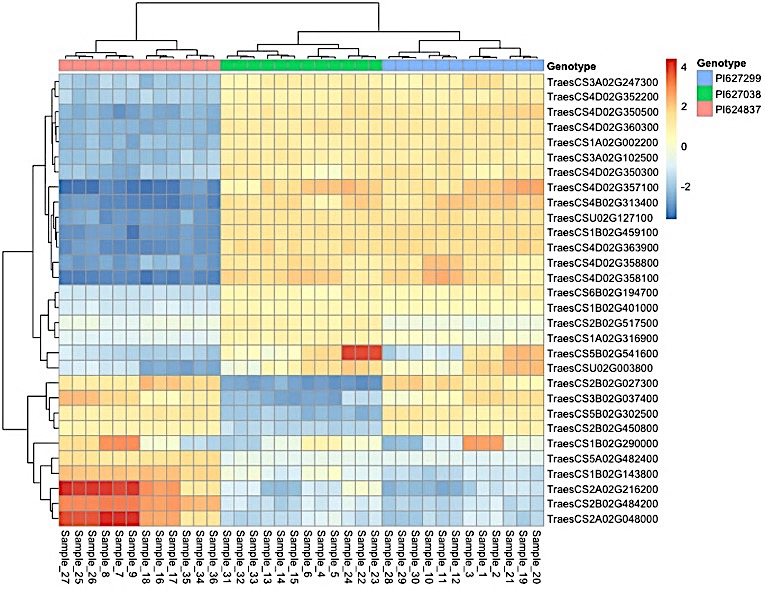


D

C

B


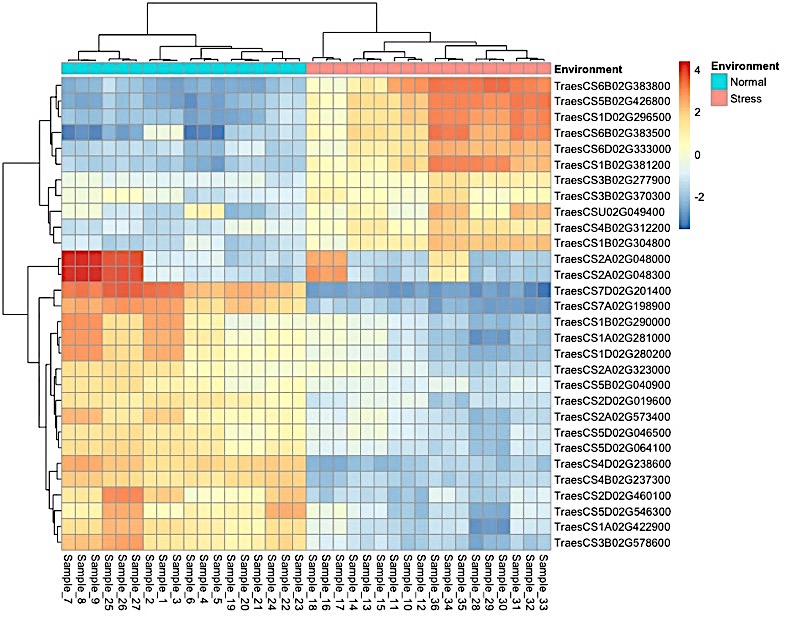

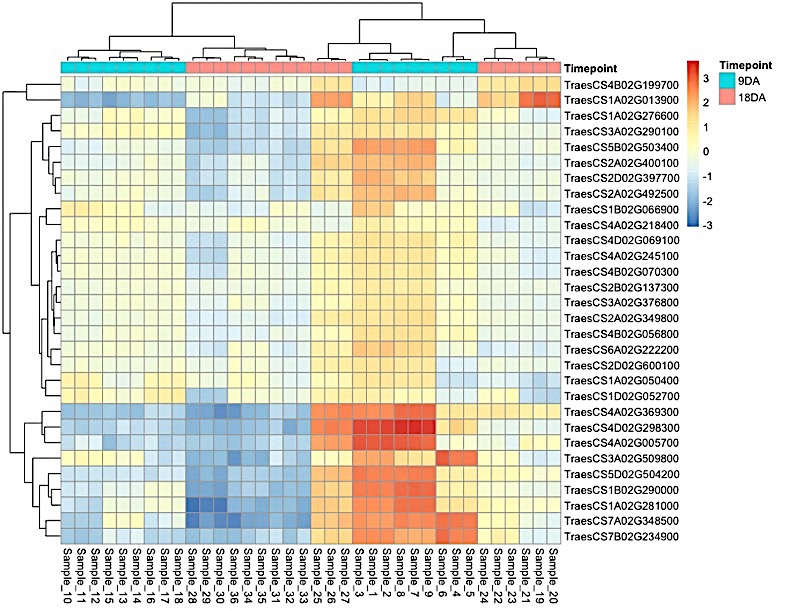


**Figure S4.** Heat map of differentially expressed genes in four comparisons. **(A)** PI627299 vs PI624837. **(B)** PI627038 vs PI624837. **(C)** Condition water deficit vs control. **(D)** Time-point 18 DA vs 9 DA. The red color represents up-regulated and blue represent down-regulated genes. Samples were clustered based on the expression level of these genes.

A


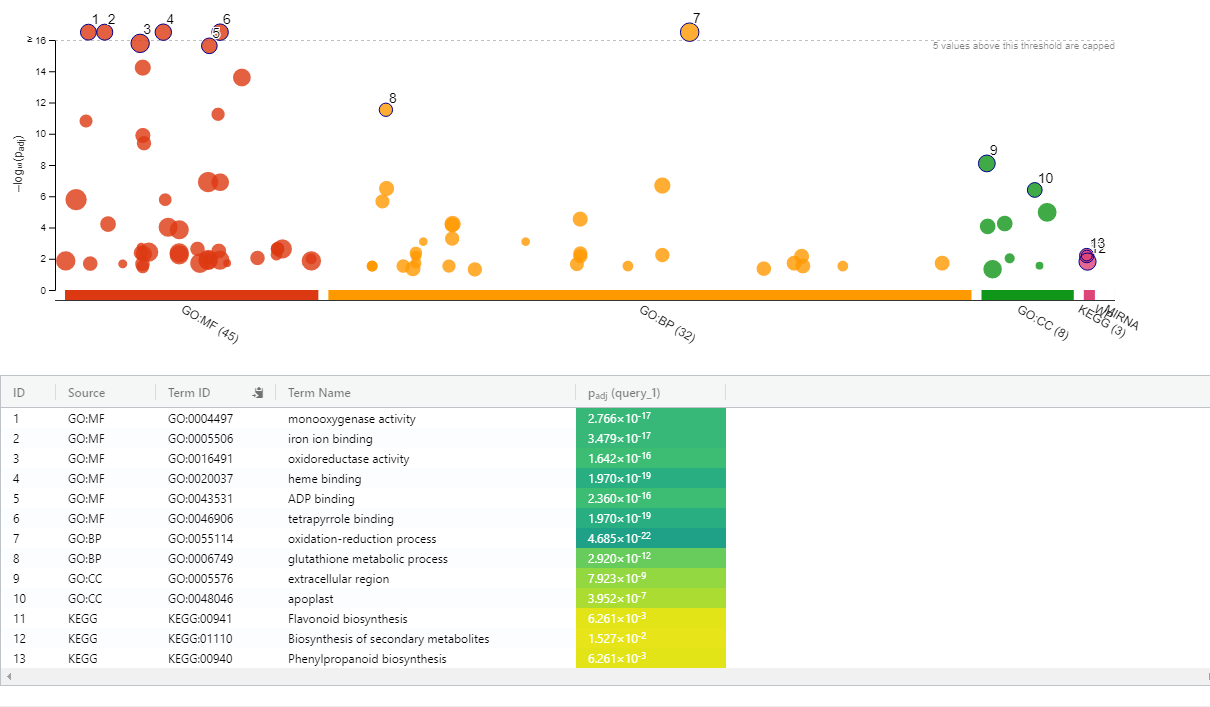


B


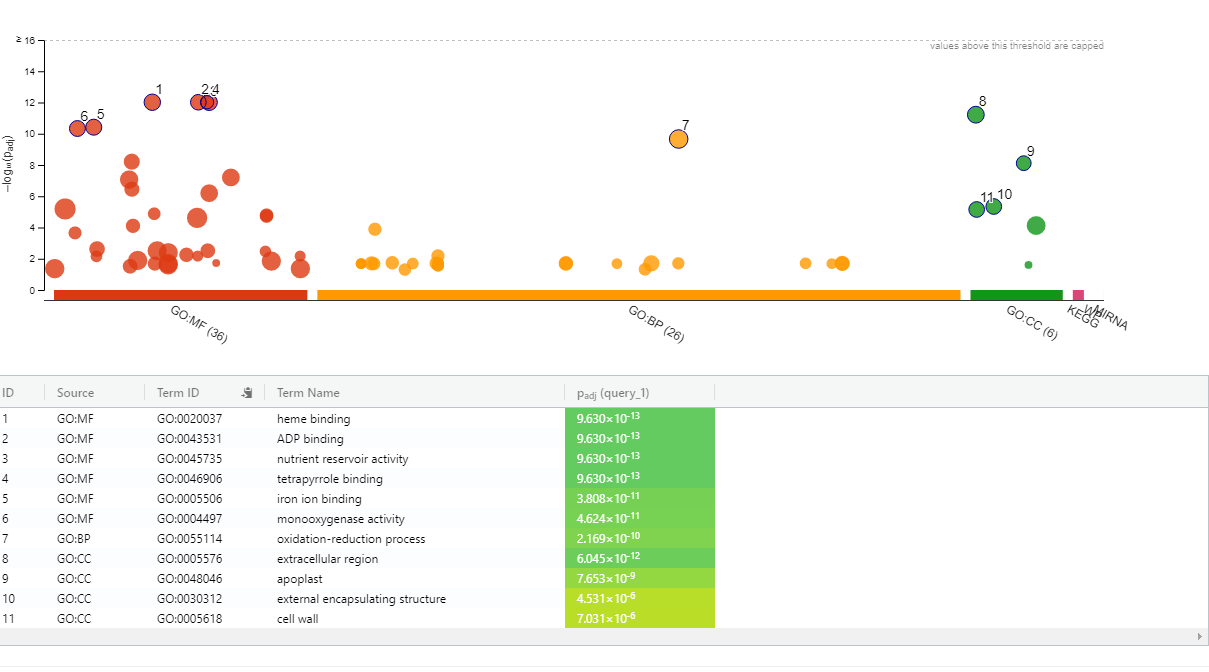


**Figure S5.** Functional analysis of DEGs on g-Profiler. **(A)** Enriched genes in comparison PI627299 vs PI624837. **(B)** Enriched genes in comparison PI627038 vs PI624837.

7
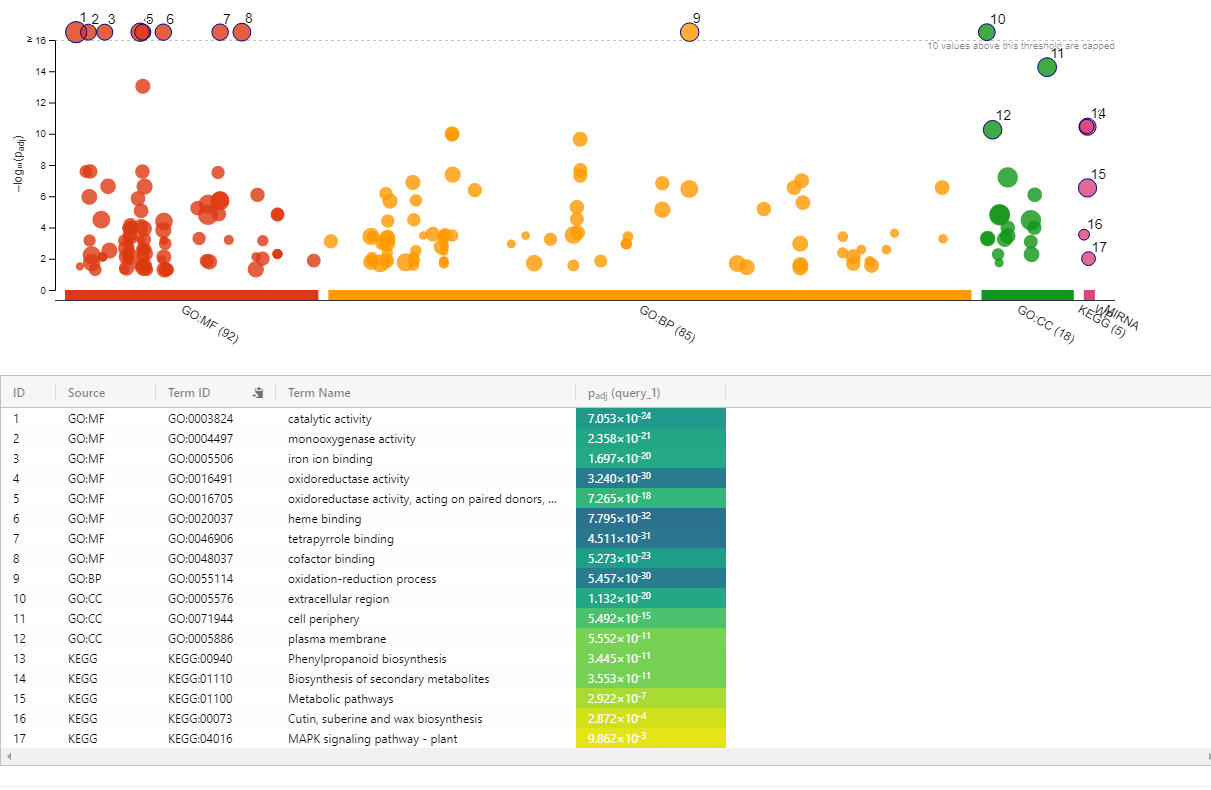


B

A


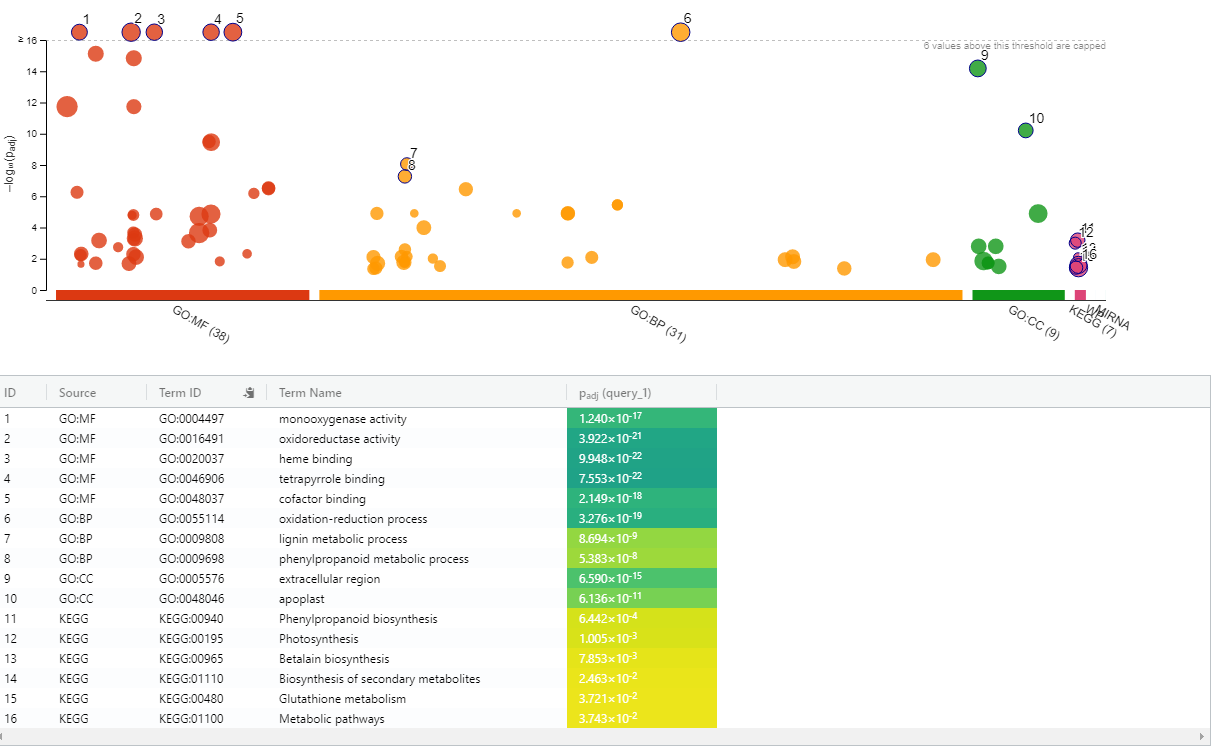


**Figure S6.** Functional analysis of DEGs on g-Profiler. **(A)** Enriched genes in comparison water deficit vs control. **(B)** Enriched genes in comparison 18DA vs 9DA.


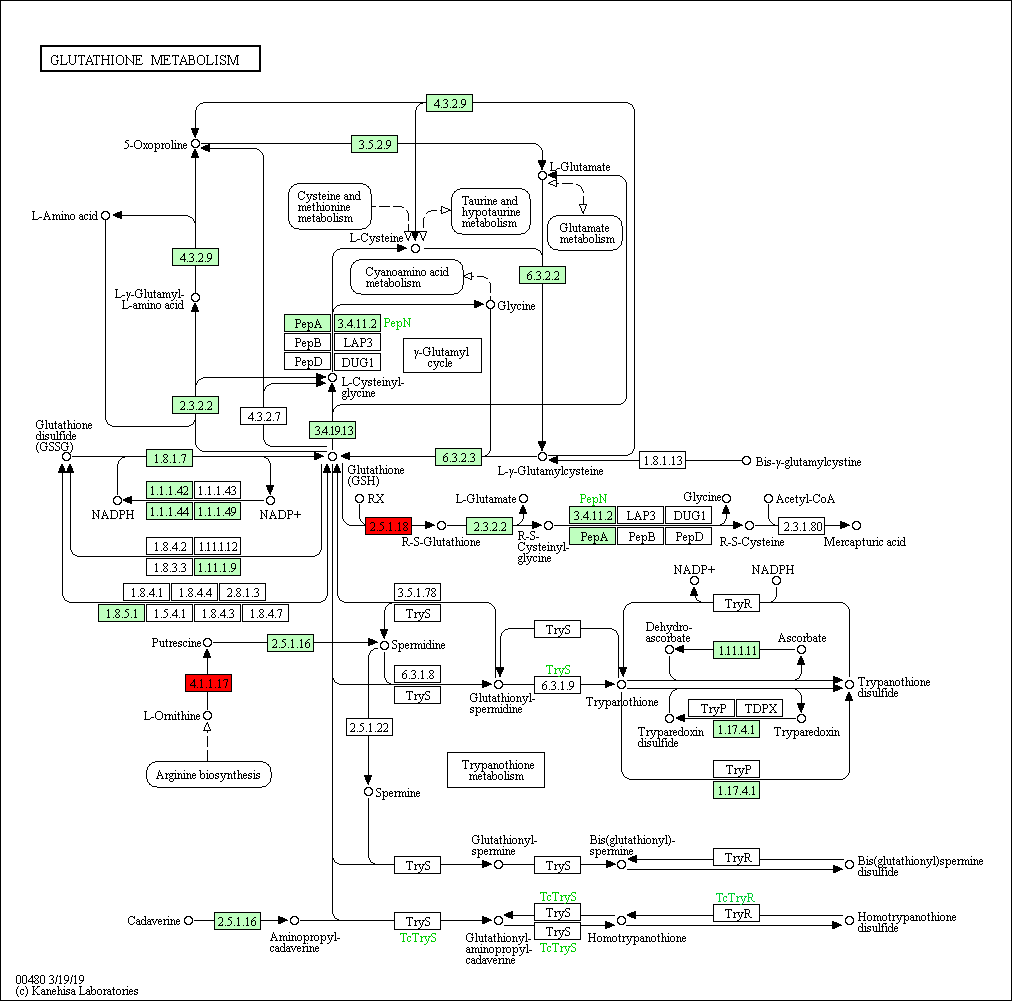


**Figure S7.** Glutathione metabolism pathway enriched in comparison water deficit vs control conditions.


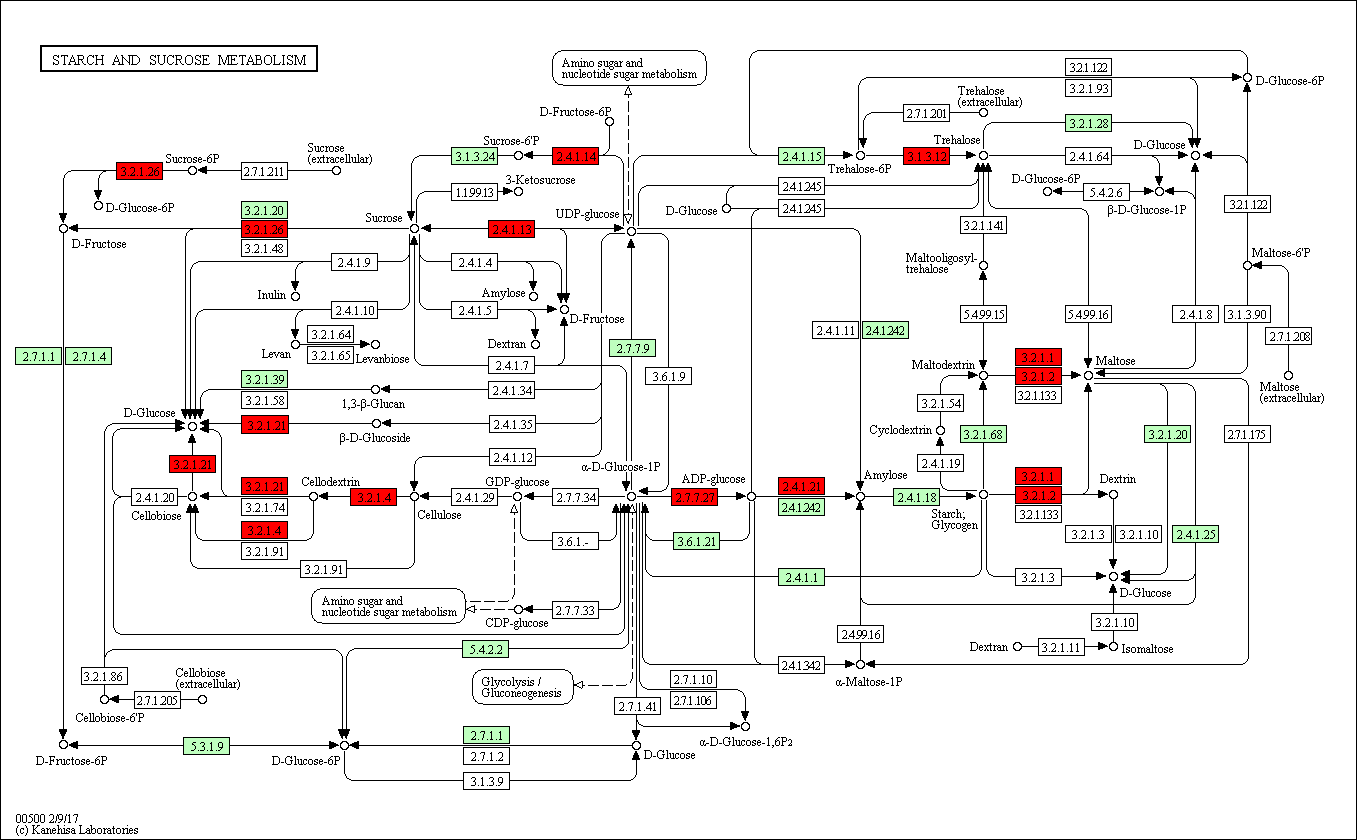


**Figure S8.** Starch and sucrose metabolism pathway enriched in comparison water deficit vs control conditions.


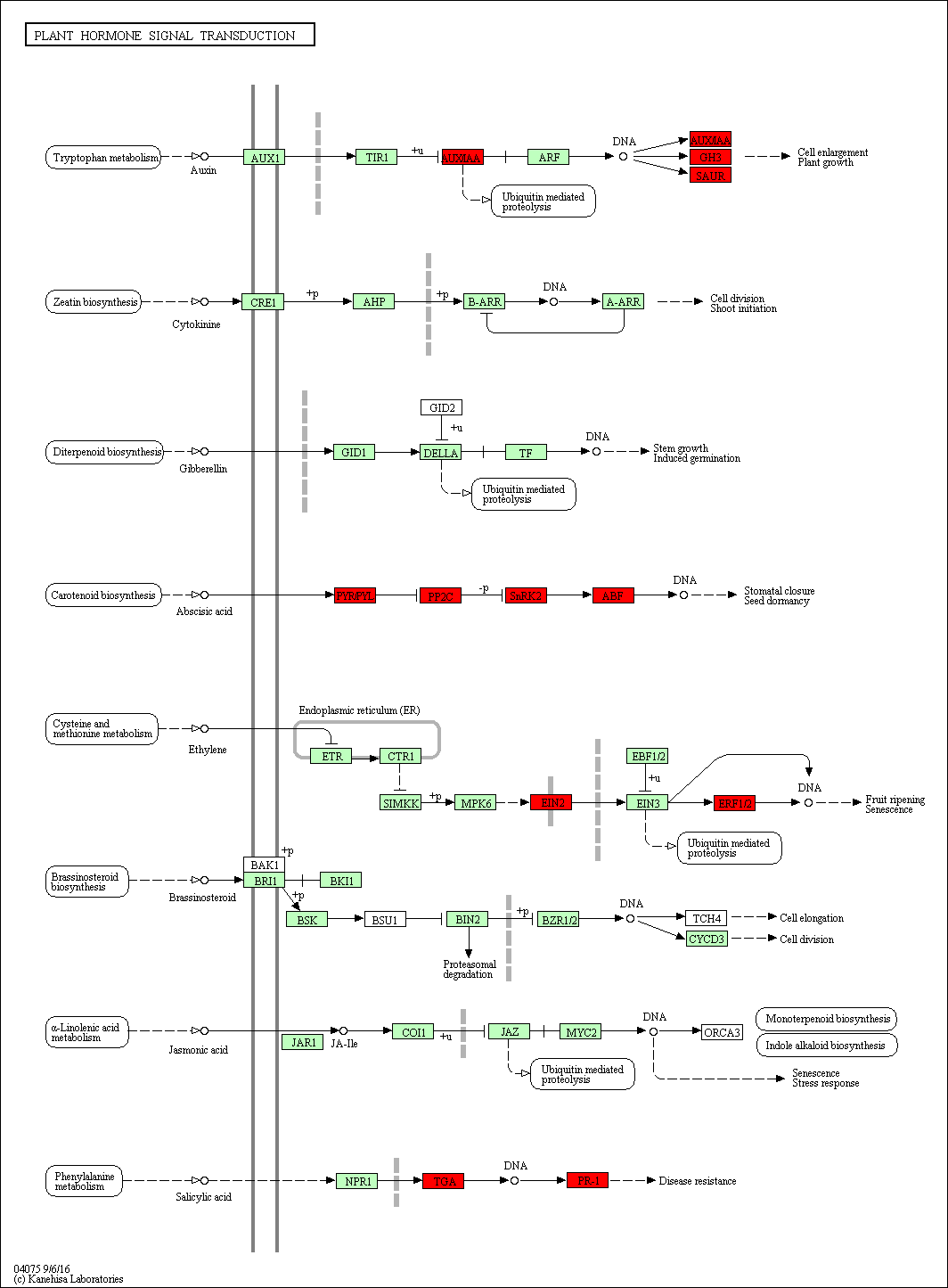


**Figure S9.** Plant hormone signal transduction enriched in comparison water deficit vs control conditions.


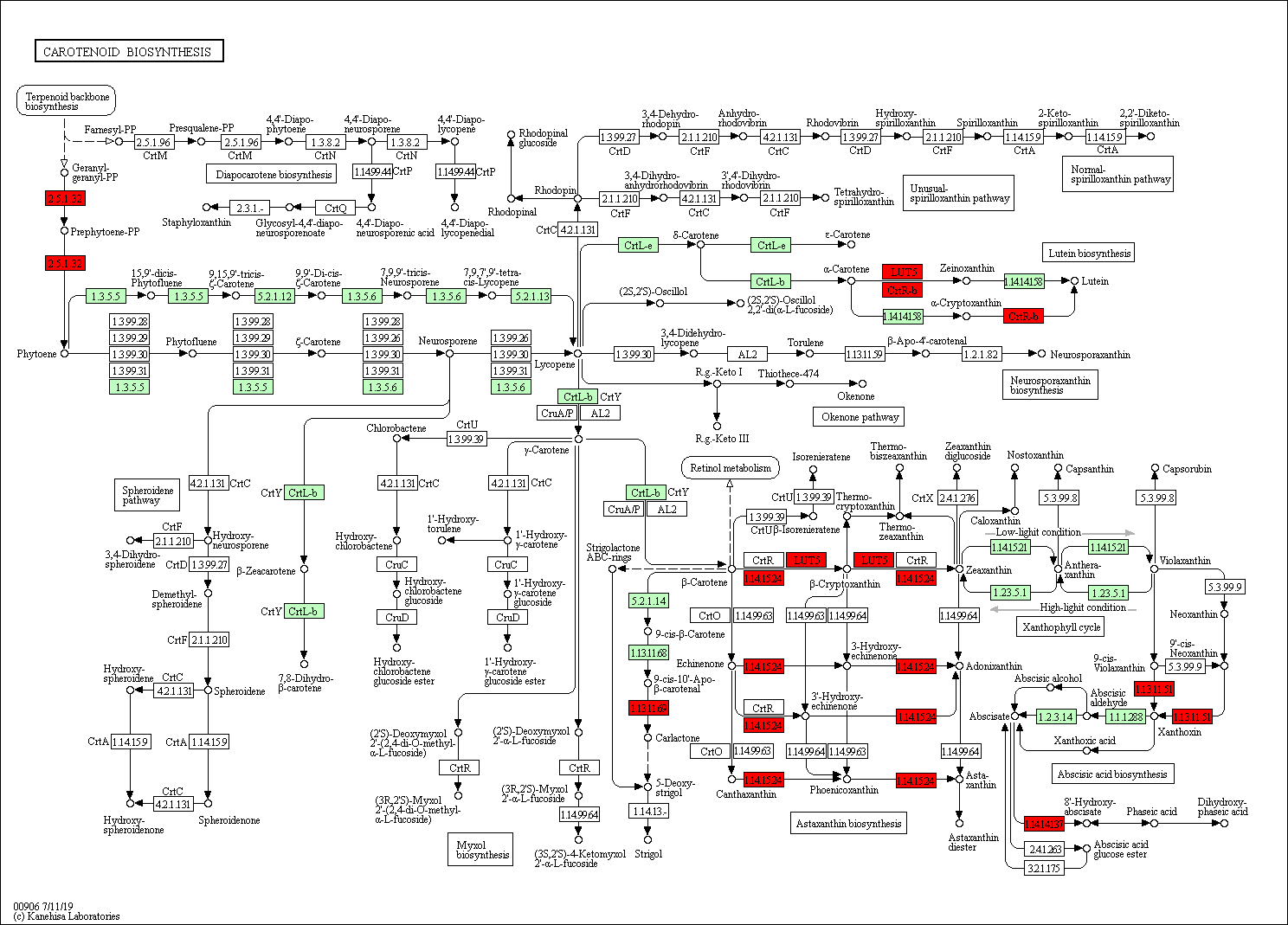


**Figure S10.** Carotenoid biosynthesis pathway enriched in comparison water deficit vs control conditions.


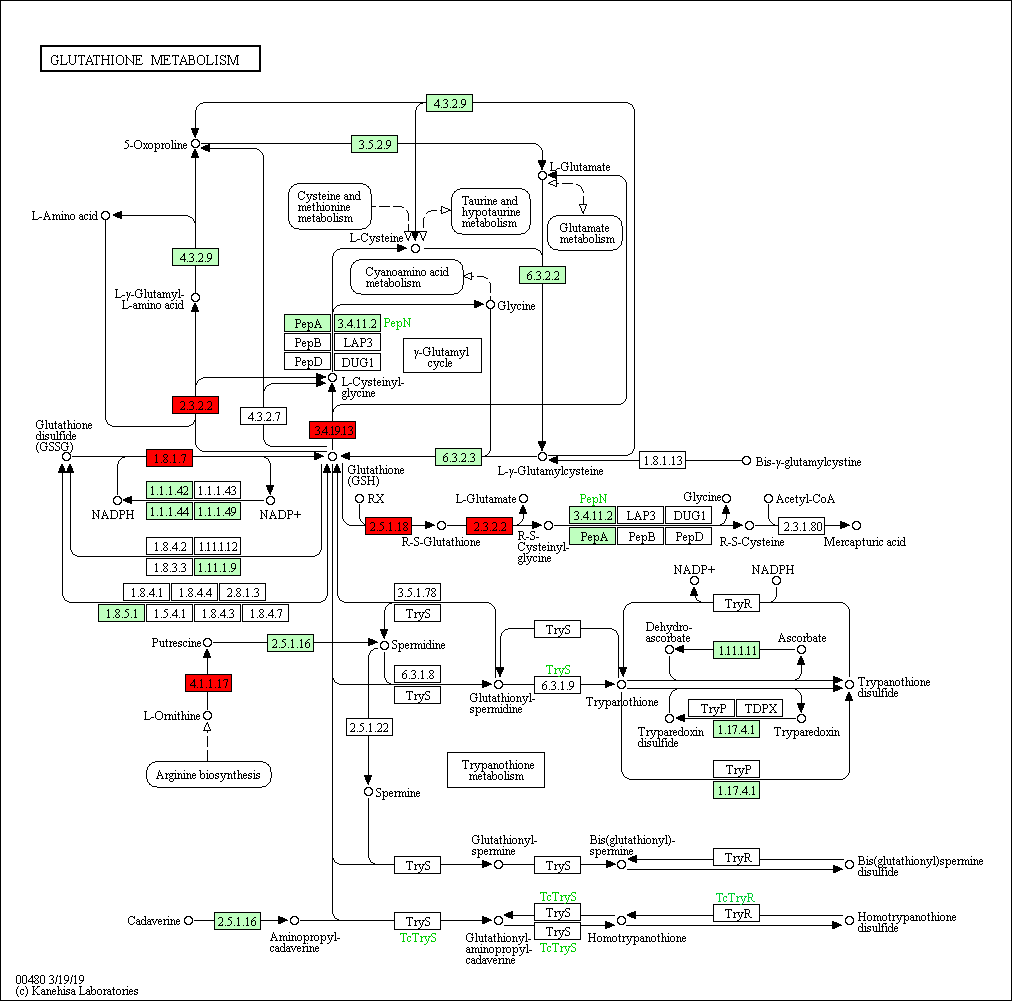


**Figure S11.** Glutathione metabolism pathway enriched in comparison PI627299 vs PI624837.


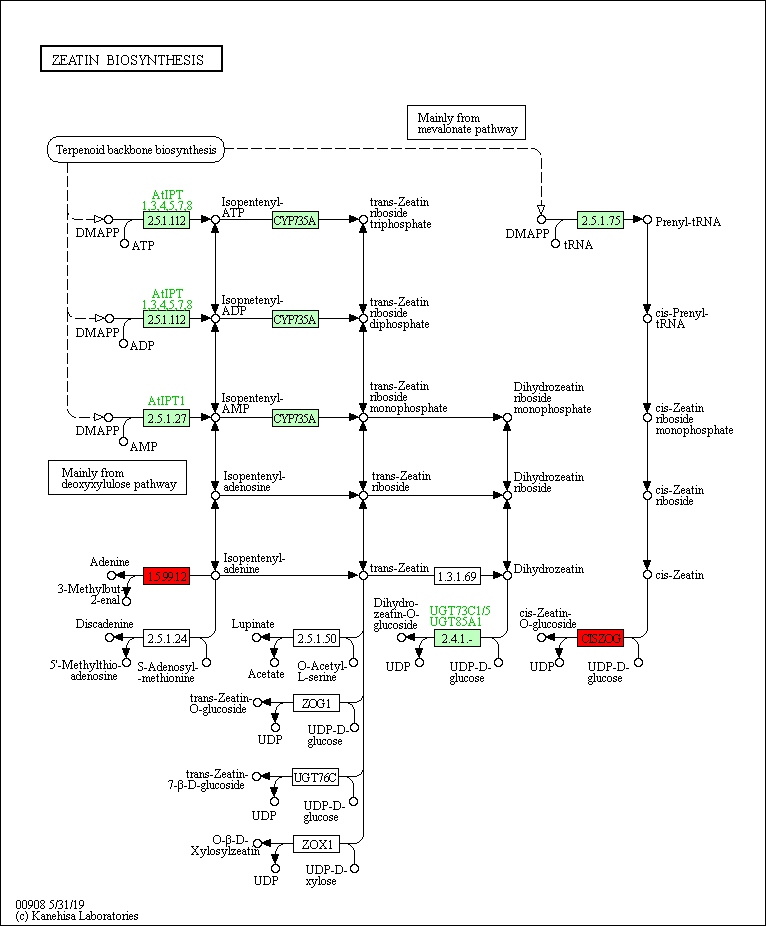


**Figure S12.** Zeatin biosynthesis pathway enriched in comparison 18DA vs 9DA.





**Figure S13.** Interaction networks of related DEGs in significant KEGG pathways and putative TFs identified by comparing PI627038 (T) vs PI624837 (S).

‘

A

| RSquare | 0.90 |
| --- | --- |
| RSquare Adj | 0.89 |
| Root Mean Square Error | 2.58 |
| Mean of Response | 1.55 |

Y = 1.16 + 0.60 (X)

RSquare Adj=0.89

B

C

**Figure S14.** Validation of differential expression data via qRT-PCR. **(A)** Linear regression pattern between the RNA-Seq log2 values and the RT-qPCR fold change. **(B)** Expression pattern of select genes, relative expression ± SD from three independent replicates in samples 9 days after stress **(B)** and samples 18 days after stress **(C)**.
