# Supplementary material for: Characterization of Dynamic Regulatory Gene and Protein Networks in Wheat Roots Upon Perceiving Water Deficit Through Comparative Transcriptomics Survey
Source: Front Plant Sci. 2021 Aug 16;12:710867. doi: 10.3389/fpls.2021.710867 (PMC8415571; doi:10.3389/fpls.2021.710867)
Supplement: Supplementary file 1 [file Data_Sheet_1.zip › Supplementary Tables S2-S8.DOCX]

**Table S2.** The list of randomly selected DEGs to was used in RT-qPCR to validate the result of RNA-seq profiling.

| **Gene ID** | **Raw** | **Primer** | **Sequence** | **Length**  **(bp)** | **Tm (°C)** | **GC**  **(%)** | **Product size**  **(bp)** |
| --- | --- | --- | --- | --- | --- | --- | --- |
| TraesCS2B02G174400 | 1 | F | AATGGACCAAGGACATCGAG | 20 | 59.9 | 50 | 199 |
|  |  | R | GACGGGTACAGGAAGCTCAG | 20 | 59.9 | 60 |  |
| TraesCS1D02G007300 | 2 | F | CGATCACTCTTGCTCCTTCC | 20 | 60 | 55 | 212 |
|  |  | R | ATTGACAGACCTCCCAGCAC | 20 | 60.1 | 55 |  |
| TraesCS2D02G587900 | 3 | F | CCGGTTGGACTCACTGATTT | 20 | 60 | 50 | 208 |
|  |  | R | GACCATCAGATCCCCTGAGA | 20 | 60 | 50 |  |
| TraesCS5B02G299700 | 4 | F | TGATGTGGATAGCAGCAAGC | 20 | 60 | 50 | 243 |
|  |  | R | CTCCCCGTTGTATGGTCACT | 20 | 59.8 | 55 |  |
| TraesCS6D02G035700 | 5 | F | TCCTTCTTCACCTGCTTCGT | 20 | 60 | 50 | 191 |
|  |  | R | AGGAAGGCACATCCATAACG | 20 | 60 | 50 |  |
| TraesCS5B02G540200 | 6 | F | GTTGGCTAGCCTTTTGCTTG | 20 | 60 | 50 | 223 |
|  |  | R | CCAAGATGCACCTGGAGATT | 20 | 60.1 | 50 |  |
| TraesCS2B02G169600 | 7 | F | GAGAAAATGCCTGCGCTTAC | 20 | 60 | 50 | 236 |
|  |  | R | TGCCGATATTGCTTCTCCTC | 20 | 60.3 | 50 |  |
| TraesCS2A02G047300 | 8 | F | ACTGGTTCACGGTGTTCCTC | 20 | 60 | 55 | 180 |
|  |  | R | CGAAGTTGGCGAAGAAGAAG | 20 | 60.1 | 50 |  |
| TraesCS2A02G051600 | 9 | F | AACACCGACATGGAGAGGTC | 20 | 60 | 55 | 167 |
|  |  | R | GCAGGAGTATCCTGGTCCAA | 20 | 60.1 | 50 |  |
| Reference (TraesCS4B02G166200) | 10 | F | AGGGCAAGCTGAGAAATTCA | 20 | 60 | 45 | 245 |
|  |  | R | ACCCAGTTCGTCATCACACA | 20 | 60 | 50 |  |

**Table S3.** Homoeologous of key genes in genotype-specific pathways on wheat chromosomes.

| **Gene** | **Species** | **Type** | **Homoeologue** | **Target %id** | **Query %id** |
| --- | --- | --- | --- | --- | --- |
| TraesCS3B02G154000 | Triticum aestivum | 1-to-1  [View Gene Tree](https://plants.ensembl.org/Triticum_aestivum/Gene/Compara_Tree?anc=31481914;db=core;g=TraesCS3B02G154000;g1=TraesCS3A02G136100;t=TraesCS3B02G154000.1) | [TraesCS3A02G136100](https://plants.ensembl.org/Triticum_aestivum/Gene/Summary?g=TraesCS3A02G136100)  [Compare Regions](https://plants.ensembl.org/Triticum_aestivum/Location/Multi?config=opt_join_genes_bottom%3Don;db=core;g=TraesCS3B02G154000;g1=TraesCS3A02G136100;r=3B:146550780-146554709;s1=Triticum_aestivum--3A;t=TraesCS3B02G154000.1) (3A:113,709,769-113,714,514:1)  [View Sequence Alignments](https://plants.ensembl.org/Triticum_aestivum/Gene/Compara_Homoeolog?db=core;g=TraesCS3B02G154000;r=3B:146550855-146554634;t=TraesCS3B02G154000.1) | 97.41 % | 97.41 % |
|  | Triticum aestivum | 1-to-1  [View Gene Tree](https://plants.ensembl.org/Triticum_aestivum/Gene/Compara_Tree?anc=31481937;db=core;g=TraesCS3B02G154000;g1=TraesCS3D02G136900;t=TraesCS3B02G154000.1) | [TraesCS3D02G136900](https://plants.ensembl.org/Triticum_aestivum/Gene/Summary?g=TraesCS3D02G136900)  [Compare Regions](https://plants.ensembl.org/Triticum_aestivum/Location/Multi?config=opt_join_genes_bottom%3Don;db=core;g=TraesCS3B02G154000;g1=TraesCS3D02G136900;r=3B:146550780-146554709;s1=Triticum_aestivum--3D;t=TraesCS3B02G154000.1) (3D:95,561,927-95,565,412:1)  [View Sequence Alignments](https://plants.ensembl.org/Triticum_aestivum/Gene/Compara_Homoeolog?db=core;g=TraesCS3B02G154000;r=3B:146550855-146554634;t=TraesCS3B02G154000.1) | 98.60 % | 98.60 % |
| TraesCS4B02G365100 | Triticum aestivum | 1-to-1  [View Gene Tree](https://plants.ensembl.org/Triticum_aestivum/Gene/Compara_Tree?anc=31481862;db=core;g=TraesCS4B02G365100;g1=TraesCS5A02G534000;t=TraesCS4B02G365100.1) | [TraesCS5A02G534000](https://plants.ensembl.org/Triticum_aestivum/Gene/Summary?g=TraesCS5A02G534000)  [Compare Regions](https://plants.ensembl.org/Triticum_aestivum/Location/Multi?config=opt_join_genes_bottom%3Don;db=core;g=TraesCS4B02G365100;g1=TraesCS5A02G534000;r=4B:653209515-653213210;s1=Triticum_aestivum--5A;t=TraesCS4B02G365100.1) (5A:691,399,290-691,403,138:-1)  [View Sequence Alignments](https://plants.ensembl.org/Triticum_aestivum/Gene/Compara_Homoeolog?db=core;g=TraesCS4B02G365100;r=4B:653209586-653213139;t=TraesCS4B02G365100.1) | 98.20 % | 98.40 % |
| TraesCS4A02G401300 | Triticum aestivum | 1-to-1  [View Gene Tree](https://plants.ensembl.org/Triticum_aestivum/Gene/Compara_Tree?anc=14918299;db=core;g=TraesCS4A02G401300;g1=TraesCS1B02G048300;t=TraesCS4A02G401300.1) | [TraesCS1B02G048300](https://plants.ensembl.org/Triticum_aestivum/Gene/Summary?g=TraesCS1B02G048300)  [Compare Regions](https://plants.ensembl.org/Triticum_aestivum/Location/Multi?config=opt_join_genes_bottom%3Don;db=core;g=TraesCS4A02G401300;g1=TraesCS1B02G048300;r=4A:675282975-675285882;s1=Triticum_aestivum--1B;t=TraesCS4A02G401300.1) (1B:28,373,087-28,375,944:-1)  [View Sequence Alignments](https://plants.ensembl.org/Triticum_aestivum/Gene/Compara_Homoeolog?db=core;g=TraesCS4A02G401300;r=4A:675283030-675285827;t=TraesCS4A02G401300.1) | 97.19 % | 97.19 % |
| TraesCS4B02G059300 | Triticum aestivum | Many-to-many  [View Gene Tree](https://plants.ensembl.org/Triticum_aestivum/Gene/Compara_Tree?anc=14029232;db=core;g=TraesCS4B02G059300;g1=TraesCS3A02G309000;t=TraesCS4B02G059300.1) | [TraesCS3A02G309000](https://plants.ensembl.org/Triticum_aestivum/Gene/Summary?g=TraesCS3A02G309000)  [Compare Regions](https://plants.ensembl.org/Triticum_aestivum/Location/Multi?config=opt_join_genes_bottom%3Don;db=core;g=TraesCS4B02G059300;g1=TraesCS3A02G309000;r=4B:50443166-50444449;s1=Triticum_aestivum--3A;t=TraesCS4B02G059300.1) (3A:547,233,557-547,235,075:-1)  [View Sequence Alignments](https://plants.ensembl.org/Triticum_aestivum/Gene/Compara_Homoeolog?db=core;g=TraesCS4B02G059300;r=4B:50443190-50444425;t=TraesCS4B02G059300.1) | 84.17 % | 95.20 % |
|  | Triticum aestivum | Many-to-many  [View Gene Tree](https://plants.ensembl.org/Triticum_aestivum/Gene/Compara_Tree?anc=14029232;db=core;g=TraesCS4B02G059300;g1=TraesCS3A02G309100;t=TraesCS4B02G059300.1) | [TraesCS3A02G309100](https://plants.ensembl.org/Triticum_aestivum/Gene/Summary?g=TraesCS3A02G309100)  [Compare Regions](https://plants.ensembl.org/Triticum_aestivum/Location/Multi?config=opt_join_genes_bottom%3Don;db=core;g=TraesCS4B02G059300;g1=TraesCS3A02G309100;r=4B:50443166-50444449;s1=Triticum_aestivum--3A;t=TraesCS4B02G059300.1) (3A:547,254,626-547,255,970:1)  [View Sequence Alignments](https://plants.ensembl.org/Triticum_aestivum/Gene/Compara_Homoeolog?db=core;g=TraesCS4B02G059300;r=4B:50443190-50444425;t=TraesCS4B02G059300.1) | 85.33 % | 96.51 % |
|  | Triticum aestivum | Many-to-many  [View Gene Tree](https://plants.ensembl.org/Triticum_aestivum/Gene/Compara_Tree?anc=14029232;db=core;g=TraesCS4B02G059300;g1=TraesCS3D02G133100;t=TraesCS4B02G059300.1) | [TraesCS3D02G133100](https://plants.ensembl.org/Triticum_aestivum/Gene/Summary?g=TraesCS3D02G133100)  [Compare Regions](https://plants.ensembl.org/Triticum_aestivum/Location/Multi?config=opt_join_genes_bottom%3Don;db=core;g=TraesCS4B02G059300;g1=TraesCS3D02G133100;r=4B:50443166-50444449;s1=Triticum_aestivum--3D;t=TraesCS4B02G059300.1) (3D:92,749,178-92,750,743:-1)  [View Sequence Alignments](https://plants.ensembl.org/Triticum_aestivum/Gene/Compara_Homoeolog?db=core;g=TraesCS4B02G059300;r=4B:50443190-50444425;t=TraesCS4B02G059300.1) | 97.82 % | 97.82 % |
|  | Triticum aestivum | Many-to-many  [View Gene Tree](https://plants.ensembl.org/Triticum_aestivum/Gene/Compara_Tree?anc=14029232;db=core;g=TraesCS4B02G059300;g1=TraesCS3D02G133200;t=TraesCS4B02G059300.1) | [TraesCS3D02G133200](https://plants.ensembl.org/Triticum_aestivum/Gene/Summary?g=TraesCS3D02G133200)  [Compare Regions](https://plants.ensembl.org/Triticum_aestivum/Location/Multi?config=opt_join_genes_bottom%3Don;db=core;g=TraesCS4B02G059300;g1=TraesCS3D02G133200;r=4B:50443166-50444449;s1=Triticum_aestivum--3D;t=TraesCS4B02G059300.1) (3D:92,783,246-92,784,549:1)  [View Sequence Alignments](https://plants.ensembl.org/Triticum_aestivum/Gene/Compara_Homoeolog?db=core;g=TraesCS4B02G059300;r=4B:50443190-50444425;t=TraesCS4B02G059300.1) | 98.25 % | 98.25 % |
| TraesCSU02G031300 | Triticum aestivum | - | - | - | - |
| TraesCS2B02G613000 | Triticum aestivum | - | - | - | - |

**Table S4.** Homoeologous of key genes in general drought-responsive pathways on wheat chromosomes.

| **Gene** | **Species** | **Type** | **Homoeologue** | **Target %id** | **Query %id** |
| --- | --- | --- | --- | --- | --- |
| TraesCS3A02G083600 | *Triticum aestivum* | 1-to-1  [View Gene Tree](https://plants.ensembl.org/Triticum_aestivum/Gene/Compara_Tree?anc=27225542;db=core;g=TraesCS3A02G083600;g1=TraesCS3D02G082600;t=TraesCS3A02G083600.1) | [TraesCS3D02G082600](https://plants.ensembl.org/Triticum_aestivum/Gene/Summary?g=TraesCS3D02G082600)  [Compare Regions](https://plants.ensembl.org/Triticum_aestivum/Location/Multi?config=opt_join_genes_bottom%3Don;db=core;g=TraesCS3A02G083600;g1=TraesCS3D02G082600;r=3A:53738543-53741548;s1=Triticum_aestivum--3D;t=TraesCS3A02G083600.1) (3D:41,782,307-41,784,592:-1)  [View Sequence Alignments](https://plants.ensembl.org/Triticum_aestivum/Gene/Compara_Homoeolog?db=core;g=TraesCS3A02G083600;r=3A:53738600-53741491;t=TraesCS3A02G083600.1) | 57.87 % | 89.18 % |
| TraesCS1B02G048300 | *Triticum aestivum* | 1-to-1  [View Gene Tree](https://plants.ensembl.org/Triticum_aestivum/Gene/Compara_Tree?anc=14918299;db=core;g=TraesCS1B02G048300;g1=TraesCS4A02G401300;t=TraesCS1B02G048300.1) | [TraesCS4A02G401300](https://plants.ensembl.org/Triticum_aestivum/Gene/Summary?g=TraesCS4A02G401300)  [Compare Regions](https://plants.ensembl.org/Triticum_aestivum/Location/Multi?config=opt_join_genes_bottom%3Don;db=core;g=TraesCS1B02G048300;g1=TraesCS4A02G401300;r=1B:28373030-28376001;s1=Triticum_aestivum--4A;t=TraesCS1B02G048300.1) (4A:675,283,030-675,285,827:-1)  [View Sequence Alignments](https://plants.ensembl.org/Triticum_aestivum/Gene/Compara_Homoeolog?db=core;g=TraesCS1B02G048300;r=1B:28373087-28375944;t=TraesCS1B02G048300.1) | 97.19 % | 97.19 % |
| TraesCS2B02G398400 | *Triticum aestivum* | 1-to-1  [View Gene Tree](https://plants.ensembl.org/Triticum_aestivum/Gene/Compara_Tree?anc=14918247;db=core;g=TraesCS2B02G398400;g1=TraesCS2A02G381100;t=TraesCS2B02G398400.1) | [TraesCS2A02G381100](https://plants.ensembl.org/Triticum_aestivum/Gene/Summary?g=TraesCS2A02G381100)  [Compare Regions](https://plants.ensembl.org/Triticum_aestivum/Location/Multi?config=opt_join_genes_bottom%3Don;db=core;g=TraesCS2B02G398400;g1=TraesCS2A02G381100;r=2B:565209719-565212608;s1=Triticum_aestivum--2A;t=TraesCS2B02G398400.1) (2A:624,458,106-624,460,956:1)  [View Sequence Alignments](https://plants.ensembl.org/Triticum_aestivum/Gene/Compara_Homoeolog?db=core;g=TraesCS2B02G398400;r=2B:565209774-565212553;t=TraesCS2B02G398400.1) | 98.32 % | 98.32 % |
|  |  | 1-to-many  [View Gene Tree](https://plants.ensembl.org/Triticum_aestivum/Gene/Compara_Tree?anc=14918248;db=core;g=TraesCS2B02G398400;g1=TraesCS2D02G377500;t=TraesCS2B02G398400.1) | [TraesCS2D02G377500](https://plants.ensembl.org/Triticum_aestivum/Gene/Summary?g=TraesCS2D02G377500)  [Compare Regions](https://plants.ensembl.org/Triticum_aestivum/Location/Multi?config=opt_join_genes_bottom%3Don;db=core;g=TraesCS2B02G398400;g1=TraesCS2D02G377500;r=2B:565209719-565212608;s1=Triticum_aestivum--2D;t=TraesCS2B02G398400.1) (2D:481,960,998-481,964,109:1)  [View Sequence Alignments](https://plants.ensembl.org/Triticum_aestivum/Gene/Compara_Homoeolog?db=core;g=TraesCS2B02G398400;r=2B:565209774-565212553;t=TraesCS2B02G398400.1) | 98.18 % | 98.18 % |
|  |  | 1-to-many  [View Gene Tree](https://plants.ensembl.org/Triticum_aestivum/Gene/Compara_Tree?anc=14918248;db=core;g=TraesCS2B02G398400;g1=TraesCS2D02G377600;t=TraesCS2B02G398400.1) | [TraesCS2D02G377600](https://plants.ensembl.org/Triticum_aestivum/Gene/Summary?g=TraesCS2D02G377600)  [Compare Regions](https://plants.ensembl.org/Triticum_aestivum/Location/Multi?config=opt_join_genes_bottom%3Don;db=core;g=TraesCS2B02G398400;g1=TraesCS2D02G377600;r=2B:565209719-565212608;s1=Triticum_aestivum--2D;t=TraesCS2B02G398400.1) (2D:481,988,232-481,990,914:1)  [View Sequence Alignments](https://plants.ensembl.org/Triticum_aestivum/Gene/Compara_Homoeolog?db=core;g=TraesCS2B02G398400;r=2B:565209774-565212553;t=TraesCS2B02G398400.1) | 98.45 % | 97.90 % |
| TraesCS2B02G398100 | *Triticum aestivum* | 1-to-many  [View Gene Tree](https://plants.ensembl.org/Triticum_aestivum/Gene/Compara_Tree?anc=14918275;db=core;g=TraesCS2B02G398100;g1=TraesCS2A02G380800;t=TraesCS2B02G398100.1) | [TraesCS2A02G380800](https://plants.ensembl.org/Triticum_aestivum/Gene/Summary?g=TraesCS2A02G380800)  [Compare Regions](https://plants.ensembl.org/Triticum_aestivum/Location/Multi?config=opt_join_genes_bottom%3Don;db=core;g=TraesCS2B02G398100;g1=TraesCS2A02G380800;r=2B:565039678-565041822;s1=Triticum_aestivum--2A;t=TraesCS2B02G398100.1) (2A:624,359,166-624,362,152:1)  [View Sequence Alignments](https://plants.ensembl.org/Triticum_aestivum/Gene/Compara_Homoeolog?db=core;g=TraesCS2B02G398100;r=2B:565039719-565041781;t=TraesCS2B02G398100.1) | 69.99 % | 95.96 % |
|  | *Triticum aestivum* | 1-to-many  [View Gene Tree](https://plants.ensembl.org/Triticum_aestivum/Gene/Compara_Tree?anc=14918273;db=core;g=TraesCS2B02G398100;g1=TraesCS2D02G377200;t=TraesCS2B02G398100.1) | [TraesCS2D02G377200](https://plants.ensembl.org/Triticum_aestivum/Gene/Summary?g=TraesCS2D02G377200)  [Compare Regions](https://plants.ensembl.org/Triticum_aestivum/Location/Multi?config=opt_join_genes_bottom%3Don;db=core;g=TraesCS2B02G398100;g1=TraesCS2D02G377200;r=2B:565039678-565041822;s1=Triticum_aestivum--2D;t=TraesCS2B02G398100.1) (2D:481,599,475-481,602,192:1)  [View Sequence Alignments](https://plants.ensembl.org/Triticum_aestivum/Gene/Compara_Homoeolog?db=core;g=TraesCS2B02G398100;r=2B:565039719-565041781;t=TraesCS2B02G398100.1) | 70.41 % | 96.54 % |
| TraesCS2B02G291100 | *Triticum aestivum* | 1-to-1  [View Gene Tree](https://plants.ensembl.org/Triticum_aestivum/Gene/Compara_Tree?anc=25574144;db=core;g=TraesCS2B02G291100;g1=TraesCS2A02G272900;t=TraesCS2B02G291100.1) | [TraesCS2A02G272900](https://plants.ensembl.org/Triticum_aestivum/Gene/Summary?g=TraesCS2A02G272900)  [Compare Regions](https://plants.ensembl.org/Triticum_aestivum/Location/Multi?config=opt_join_genes_bottom%3Don;db=core;g=TraesCS2B02G291100;g1=TraesCS2A02G272900;r=2B:404204801-404207177;s1=Triticum_aestivum--2A;t=TraesCS2B02G291100.1) (2A:447,003,956-447,006,094:-1)  [View Sequence Alignments](https://plants.ensembl.org/Triticum_aestivum/Gene/Compara_Homoeolog?db=core;g=TraesCS2B02G291100;r=2B:404204846-404207132;t=TraesCS2B02G291100.1) | 99.35 % | 99.52 % |
|  | *Triticum aestivum* | 1-to-1  [View Gene Tree](https://plants.ensembl.org/Triticum_aestivum/Gene/Compara_Tree?anc=25574143;db=core;g=TraesCS2B02G291100;g1=TraesCS2D02G272200;t=TraesCS2B02G291100.1) | [TraesCS2D02G272200](https://plants.ensembl.org/Triticum_aestivum/Gene/Summary?g=TraesCS2D02G272200)  [Compare Regions](https://plants.ensembl.org/Triticum_aestivum/Location/Multi?config=opt_join_genes_bottom%3Don;db=core;g=TraesCS2B02G291100;g1=TraesCS2D02G272200;r=2B:404204801-404207177;s1=Triticum_aestivum--2D;t=TraesCS2B02G291100.1) (2D:336,734,932-336,737,192:-1)  [View Sequence Alignments](https://plants.ensembl.org/Triticum_aestivum/Gene/Compara_Homoeolog?db=core;g=TraesCS2B02G291100;r=2B:404204846-404207132;t=TraesCS2B02G291100.1) | 99.52 % | 99.52 % |

**Table S4. Continued.** Homoeologous of key genes in general drought-responsive pathways on wheat chromosomes.

| **Gene** | **Species** | **Type** | **Homoeologue** | **Target %id** | **Query %id** |
| --- | --- | --- | --- | --- | --- |
| TraesCS6D02G248000 | *Triticum aestivum* | 1-to-1  [View Gene Tree](https://plants.ensembl.org/Triticum_aestivum/Gene/Compara_Tree?anc=26365494;db=core;g=TraesCS6D02G248000;g1=TraesCS6A02G266700;t=TraesCS6D02G248000.1) | [TraesCS6A02G266700](https://plants.ensembl.org/Triticum_aestivum/Gene/Summary?g=TraesCS6A02G266700)  [Compare Regions](https://plants.ensembl.org/Triticum_aestivum/Location/Multi?config=opt_join_genes_bottom%3Don;db=core;g=TraesCS6D02G248000;g1=TraesCS6A02G266700;r=6D:351134297-351138648;s1=Triticum_aestivum--6A;t=TraesCS6D02G248000.1) (6A:492,282,909-492,286,285:-1)  [View Sequence Alignments](https://plants.ensembl.org/Triticum_aestivum/Gene/Compara_Homoeolog?db=core;g=TraesCS6D02G248000;r=6D:351134380-351138565;t=TraesCS6D02G248000.1) | 97.03 % | 97.89 % |
|  | *Triticum aestivum* | 1-to-1  [View Gene Tree](https://plants.ensembl.org/Triticum_aestivum/Gene/Compara_Tree?anc=26365495;db=core;g=TraesCS6D02G248000;g1=TraesCS6B02G294100;t=TraesCS6D02G248000.1) | [TraesCS6B02G294100](https://plants.ensembl.org/Triticum_aestivum/Gene/Summary?g=TraesCS6B02G294100)  [Compare Regions](https://plants.ensembl.org/Triticum_aestivum/Location/Multi?config=opt_join_genes_bottom%3Don;db=core;g=TraesCS6D02G248000;g1=TraesCS6B02G294100;r=6D:351134297-351138648;s1=Triticum_aestivum--6B;t=TraesCS6D02G248000.1) (6B:528,284,752-528,288,193:-1)  [View Sequence Alignments](https://plants.ensembl.org/Triticum_aestivum/Gene/Compara_Homoeolog?db=core;g=TraesCS6D02G248000;r=6D:351134380-351138565;t=TraesCS6D02G248000.1) | 97.38 % | 98.24 % |
| TraesCS1D02G039500 | *Triticum aestivum* | 1-to-1  [View Gene Tree](https://plants.ensembl.org/Triticum_aestivum/Gene/Compara_Tree?anc=14918286;db=core;g=TraesCS1D02G039500;g1=TraesCS1A02G037800;t=TraesCS1D02G039500.1) | [TraesCS1A02G037800](https://plants.ensembl.org/Triticum_aestivum/Gene/Summary?g=TraesCS1A02G037800)  [Compare Regions](https://plants.ensembl.org/Triticum_aestivum/Location/Multi?config=opt_join_genes_bottom%3Don;db=core;g=TraesCS1D02G039500;g1=TraesCS1A02G037800;r=1D:19022308-19025067;s1=Triticum_aestivum--1A;t=TraesCS1D02G039500.1) (1A:20,935,423-20,938,081:1)  [View Sequence Alignments](https://plants.ensembl.org/Triticum_aestivum/Gene/Compara_Homoeolog?db=core;g=TraesCS1D02G039500;r=1D:19022361-19025014;t=TraesCS1D02G039500.1) | 98.32 % | 98.32 % |
|  | *Triticum aestivum* | 1-to-1  [View Gene Tree](https://plants.ensembl.org/Triticum_aestivum/Gene/Compara_Tree?anc=14918286;db=core;g=TraesCS1D02G039500;g1=TraesCS1B02G048500;t=TraesCS1D02G039500.1) | [TraesCS1B02G048500](https://plants.ensembl.org/Triticum_aestivum/Gene/Summary?g=TraesCS1B02G048500)  [Compare Regions](https://plants.ensembl.org/Triticum_aestivum/Location/Multi?config=opt_join_genes_bottom%3Don;db=core;g=TraesCS1D02G039500;g1=TraesCS1B02G048500;r=1D:19022308-19025067;s1=Triticum_aestivum--1B;t=TraesCS1D02G039500.1) (1B:28,483,566-28,486,349:1)  [View Sequence Alignments](https://plants.ensembl.org/Triticum_aestivum/Gene/Compara_Homoeolog?db=core;g=TraesCS1D02G039500;r=1D:19022361-19025014;t=TraesCS1D02G039500.1) | 98.18 % | 98.18 % |
| TraesCS6A02G041800 | *Triticum aestivum* | 1-to-1  [View Gene Tree](https://plants.ensembl.org/Triticum_aestivum/Gene/Compara_Tree?anc=23130589;db=core;g=TraesCS6A02G041800;g1=TraesCS6D02G004300;t=TraesCS6A02G041800.1) | [TraesCS6D02G004300](https://plants.ensembl.org/Triticum_aestivum/Gene/Summary?g=TraesCS6D02G004300)  [Compare Regions](https://plants.ensembl.org/Triticum_aestivum/Location/Multi?config=opt_join_genes_bottom%3Don;db=core;g=TraesCS6A02G041800;g1=TraesCS6D02G004300;r=6A:22070839-22072066;s1=Triticum_aestivum--6D;t=TraesCS6A02G041800.1) (6D:2,106,676-2,108,293:-1)  [View Sequence Alignments](https://plants.ensembl.org/Triticum_aestivum/Gene/Compara_Homoeolog?db=core;g=TraesCS6A02G041800;r=6A:22070862-22072043;t=TraesCS6A02G041800.1) | 79.00 % | 88.04 % |
|  | *Triticum aestivum* | 1-to-many  [View Gene Tree](https://plants.ensembl.org/Triticum_aestivum/Gene/Compara_Tree?anc=23130585;db=core;g=TraesCS6A02G041800;g1=TraesCS6B02G006200;t=TraesCS6A02G041800.1) | [TraesCS6B02G006200](https://plants.ensembl.org/Triticum_aestivum/Gene/Summary?g=TraesCS6B02G006200)  [Compare Regions](https://plants.ensembl.org/Triticum_aestivum/Location/Multi?config=opt_join_genes_bottom%3Don;db=core;g=TraesCS6A02G041800;g1=TraesCS6B02G006200;r=6A:22070839-22072066;s1=Triticum_aestivum--6B;t=TraesCS6A02G041800.1) (6B:4,451,488-4,453,061:1)  [View Sequence Alignments](https://plants.ensembl.org/Triticum_aestivum/Gene/Compara_Homoeolog?db=core;g=TraesCS6A02G041800;r=6A:22070862-22072043;t=TraesCS6A02G041800.1) | 79.09 % | 88.55 % |
| TraesCS6A02G001500 | *Triticum aestivum* | 1-to-many  [View Gene Tree](https://plants.ensembl.org/Triticum_aestivum/Gene/Compara_Tree?anc=23130585;db=core;g=TraesCS6A02G001500;g1=TraesCS6B02G006200;t=TraesCS6A02G001500.1) | [TraesCS6B02G006200](https://plants.ensembl.org/Triticum_aestivum/Gene/Summary?g=TraesCS6B02G006200)  [Compare Regions](https://plants.ensembl.org/Triticum_aestivum/Location/Multi?config=opt_join_genes_bottom%3Don;db=core;g=TraesCS6A02G001500;g1=TraesCS6B02G006200;r=6A:786700-788346;s1=Triticum_aestivum--6B;t=TraesCS6A02G001500.1) (6B:4,451,488-4,453,061:1)  [View Sequence Alignments](https://plants.ensembl.org/Triticum_aestivum/Gene/Compara_Homoeolog?db=core;g=TraesCS6A02G001500;r=6A:786731-788315;t=TraesCS6A02G001500.1) | 87.50 % | 87.90 % |
| TraesCS2B02G613000 | *Triticum aestivum* | - | - | - | - |

**Table S5.** A summary of identified transcriptional regulators among different collection of DEGs in wheat.

| Comparison | Transcriptional Regulators | | | | |
| --- | --- | --- | --- | --- | --- |
| PI627299 vs PI624837 | Coactivator p15 (1) | GNAT (1) | mTERF (15) | Others (11) | PHD (1) |
|  | SET (1) | SNF2 (5) | SWI/SNF-SW13 (1) | SWI/SNF-BAF60b (2) | TAZ (4) |
|  | TRAF (5) |  |  |  |  |
| PI627038 vs PI624837 | Coactivator p15 (2) | mTERF (10) | Others (2) | SET (1) | SNF2 (1) |
|  | SWI/SNF-SWI3 (1) | SWI/SNF-BAF60B (1) | TAZ (3) | TRAF (8) |  |
| Water deficit vs Control | AUX/IAA (2) | Others (6) | PHD (2) | Rcd1-like (3) | SNF2 (1) |
|  | TAZ (8) |  |  |  |  |
| 18 DA vs 9 DA | MBF1 (3) | mTERF (1) | Others (10) | SNF2 (1) |  |

**Table S6.** The list of KEGG pathways significantly enriched for the DEGs at lines comparison.

| Comparison | KEGG pathway | Corrected p-value | *Triticum aestivum* genes |
| --- | --- | --- | --- |
| PI627299 vs PI624837 | Glutathione metabolism | 0.002 | TraesCS3B02G571200\|TraesCS7A02G531100\|TraesCS3D02G133100\|TraesCS2A02G578300\|TraesCS2D02G579500\|TraesCS3B02G571200\|TraesCS5A02G424100\|TraesCS3A02G452900\|TraesCS7A02G531100\|TraesCS6B02G423000\|TraesCS3A02G076700\|TraesCS4D02G201100\|TraesCS5B02G304200 |
|  | Flavonoid biosynthesis | 0.003 | TraesCS2B02G508300\|TraesCS2B02G508300\|TraesCSU02G138300\|TraesCS6A02G012600\|TraesCS2B02G182500 |
| PI627038 vs PI624837 | Glutathione metabolism | 0.0009 | TraesCS3B02G571100\|TraesCS3B02G471600\|TraesCS3B02G152200\|TraesCS2A02G578300\|TraesCS2D02G579500\|TraesCS3B02G471600\|TraesCS2D02G579500\|TraesCS3B02G571100\|TraesCS5A02G424100\|TraesCS3A02G452900\|TraesCS5B02G304200\|TraesCS4D02G201100  TraesCS5B02G304200\|TraesCS6B02G423000\|TraesCS3A02G076700 |
|  | Valine, leucine and isoleucine degradation | 0.016 | TraesCS1B02G085100\|TraesCS6A02G036000\|TraesCS2A02G036300\|TraesCS2A02G036300\|  TraesCS4D02G356200\|TraesCS4D02G170300\|TraesCS4B02G235400\|TraesCS4B02G071500\|TraesCS4B02G071500 |
|  | Flavonoid biosynthesis | 0.016 | TraesCS2D02G500300\|TraesCS2D02G500300\|TraesCSU02G138300\|TraesCS6D02G015200\|  TraesCS2B02G182500\|TraesCS2B02G182500\| |
|  | Phenylpropanoid biosynthesis | 0.034 | TraesCS2A02G573600\|TraesCS2B02G124600\|TraesCS1B02G024000\|TraesCS7B02G378700\|TraesCS1A02G318300\|TraesCSU02G138300\|TraesCS2D02G108200\|TraesCS2B02G182500\|TraesCS1B02G115000\|TraesCS2A02G509800\|TraesCS2B02G182500 |

**Table S7.** The list of KEGG pathways significantly enriched for the DEGs at conditions comparison.

| Comparison | KEGG pathway | Corrected p-value | *Triticum aestivum* genes |
| --- | --- | --- | --- |
| Water deficit vs Control | Phenylpropanoid biosynthesis | 6.92E-09 | TraesCS2A02G329000\|TraesCS1D02G019400\|TraesCS5A02G388500\|TraesCS7D02G184500\|TraesCS2A02G355800\|TraesCS2D02G108100\|TraesCS1A02G104100\|TraesCS5D02G399100\|TraesCS2A02G573400\|TraesCS2B02G174400\|TraesCS5A02G399500\|TraesCS1B02G331100\|TraesCS1B02G048200\|TraesCS2B02G291100\|TraesCS3A02G509600\|TraesCS2A02G509800\|TraesCS7A02G353300\|TraesCS4A02G196300\|TraesCS2B02G124600\|TraesCS5A02G295100\|TraesCS1B02G115900\|TraesCS2D02G353000\|TraesCS1A02G108400\|TraesCS6A02G266700\|TraesCS2B02G182500  TraesCS5A02G400500\|TraesCS4A02G196300\|TraesCS1A02G356100\|TraesCS7D02G184500\|TraesCS7B02G378700\|TraesCS2B02G174400\|TraesCS2B02G182500\|TraesCS2B02G084500\|TraesCS2A02G107300\|TraesCS3A02G509600\|TraesCS2A02G329000 |
|  | Flavonoid biosynthesis | 0.011 | TraesCS2A02G482200\|TraesCS6D02G004300\|TraesCS4B02G344800\|TraesCS2A02G482200\|TraesCS2B02G182500\|TraesCS6D02G004300\|TraesCS2B02G182500 |
|  | Glutathione metabolism | 0.017 | TraesCS1D02G190200\|TraesCS3A02G309000\|TraesCS3A02G376700\|TraesCS1D02G190200\|TraesCS1A02G186700\|TraesCS2A02G578300\|TraesCS1A02G186700\|TraesCS3A02G452900\|TraesCS7D02G156900\|TraesCS1D02G095100\|TraesCS7D02G156900\|TraesCS3B02G538500\|TraesCS4A02G104300\|TraesCS4A02G008400 |
|  |  |  |  |

**Table S7. continued.** The list of KEGG pathways significantly enriched for the DEGs at conditions comparison.

| Comparison | KEGG pathway | Corrected p-value | *Triticum aestivum* genes |
| --- | --- | --- | --- |
| Water deficit vs Control | Starch and sucrose metabolism | 0.022 | TraesCS2A02G329000\|TraesCS1D02G264000\|TraesCS5A02G281600\|TraesCS5A02G388500\|TraesCS5B02G175800\|TraesCS3A02G226400\|TraesCS5D02G399100\|TraesCS7B02G286700\|TraesCS2A02G161100\|TraesCS2A02G489100\|TraesCS7A02G180800\|TraesCSU02G044500\|TraesCS5D02G323700\|TraesCS5A02G295100\|TraesCS1B02G243000\|TraesCS2D02G099900\|TraesCS7A02G009200\|TraesCS4B02G344300\|TraesCS5A02G043800\|TraesCS4D02G169800\|TraesCS2A02G168200\|TraesCS2A02G329000\|TraesCS7A02G189000 |
|  | Plant hormone signal transduction | 0.030 | TraesCS2D02G383200\|TraesCS1B02G306900\|TraesCS5A02G183600\|TraesCS3A02G378700\|TraesCS2A02G493800\|TraesCS4A02G094300\|TraesCS4D02G191200\|TraesCS1A02G411200  TraesCS1B02G281100\|TraesCS1A02G358600\|TraesCS4A02G235600\|TraesCS3B02G353200\|TraesCSU02G095300\|TraesCS1A02G276600\|TraesCS2B02G133000\|TraesCS4A02G204900\|TraesCS3A02G154400\|TraesCS2A02G494300\|TraesCS3A02G237800\|TraesCS1A02G328800\|TraesCS2B02G437000\|TraesCS7A02G198800\|TraesCS3A02G437000\|TraesCS7A02G170600\|TraesCS5A02G439700 |
|  | Carotenoid biosynthesis | 0.05 | TraesCS4B02G318000\|TraesCS5D02G383500\|TraesCS5D02G244900\|TraesCS2A02G250600\|TraesCS5A02G356300\|TraesCS6B02G438800\|TraesCS3A02G274300 |

**Table S8.** The list of KEGG pathways significantly enriched for the DEGs at time-points comparison.

| Comparison | KEGG pathway | Corrected p-value | *Triticum aestivum* genes |
| --- | --- | --- | --- |
| 18 DA vs 9 DA | Glutathione metabolism | 0.001 | TraesCS1D02G190200\|TraesCS3A02G309000\|TraesCS1A02G186700\|TraesCS1A02G186700\|TraesCS1D02G190200\|TraesCS1D02G095100\|TraesCS4A02G008400\|TraesCS7D02G156900\|TraesCS3B02G538500\|TraesCS4A02G104300\|TraesCS7D02G156900 |
|  | Zeatin biosynthesis | 0.016 | TraesCS2B02G428700\|TraesCS3A02G481000\|TraesCS3A02G321100\|TraesCS2B02G428700 |
|  | Photosynthesis | 0.020 | TraesCS7D02G311300\|TraesCS2A02G247300\|TraesCS2B02G312600\|TraesCS6A02G374400\|TraesCS2B02G240000\|TraesCS2D02G255100\|TraesCS2B02G272300\|TraesCS4B02G170800 |
|  | Phenylpropanoid biosynthesis | 0.023 | TraesCS2B02G124600\|TraesCS3B02G061000\|TraesCS6A02G222700\|TraesCS2A02G355300\|TraesCS2B02G182500\|TraesCS1B02G048500  TraesCSU02G062800\|TraesCS2A02G107300\|TraesCS2A02G108000\|TraesCS2B02G182500\|TraesCSU02G062800 |
|  | Flavonoid biosynthesis | 0.029 | TraesCS2B02G182500\|TraesCS6D02G004300\|TraesCS6D02G004300\|TraesCS2B02G182500 |
